# Depletion of CD8^+^ CART cells leads to superior anti-tumor efficacy of pure CD4^+^ CART cells against acute leukemias

**DOI:** 10.1016/j.ymthe.2026.05.027

**Published:** 2026-06-03

**Authors:** Qian Chen, David Sedloev, Hao Yao, Lianghao Mao, Dominic Depke, Lei Wang, Marina Scheller, Bailin He, Yi Liu, Julia M. Unglaub, Anita Schmitt, Ashok Kumar Jayavelu, Carsten Müller-Tidow, Michael Schmitt, Tim Sauer

**Affiliations:** 1 Department of Medicine V, Hematology, Oncology and Rheumatology, University Hospital Heidelberg, 69120 Heidelberg, Germany; 2 Department of Lymphoma, Zhejiang Cancer Hospital, Hangzhou Institute of Medicine (HIM), Chinese Academy of Sciences, Hangzhou 310022, China; 3 Proteomics and Cancer Cell Signaling Group, Clinical Cooperation Unit Pediatric Leukemia, German Cancer Research Center and Department of Pediatric Oncology, Hematology and Immunology, University of Heidelberg, 69120 Heidelberg, Germany; 4 Department of Hematology, Nanfang Hospital, Southern Medical University, Guangzhou 510515, China; 5 Hopp Children’s Cancer Center, 69120 Heidelberg, Germany

**Keywords:** CD4^+^CART, CD8^+^ CART, acute leukemia, CD70.CART, CD19.CART, CD33.CART

## Abstract

Chimeric antigen receptor T (CART) cell therapy has demonstrated clinical efficacy in hematologic malignancies; however, primary or secondary treatment failure remains a major obstacle to durable responses. Defining the optimal cellular composition of CART products therefore represents a critical unmet need. Here, we show that CARTs targeting acute leukemias achieve maximal efficacy when composed exclusively of CD4^+^ T cells. Pure CD4^+^ CARTs exhibit superior anti-tumor activity and proliferation compared with CD8^+^-containing CART products. To elucidate the molecular basis of this functional divergence, we applied a combinatorial exploratory approach integrating bulk RNA sequencing and quantitative proteomics. Transcriptomic analyses revealed that pure CD4^+^ CARTs adopt a highly proliferative state characterized by a cytotoxic effector-like polarization. On the protein level, we are demonstrating coordinated loss of cell-cycle machinery and induction of apoptotic pathways in CD4^+^ CART cells co-cultured together with CD8^+^ CART cells, while pure CD4^+^ CART cultures maintained a proliferative, cytotoxic phenotype. Using mechanistically discriminative co-culture systems, we demonstrate that CD8^+^ CART-mediated impairment of CD4^+^ CART functionality is primarily driven by competitive access to shared antigen. Collectively, these findings identify antigen competition between CART subsets as a previously unrecognized mechanism limiting CD4^+^ CART efficacy and provide a framework for optimizing CART product composition to enhance therapeutic persistence and durability.

## Graphical abstract

**Figure:**
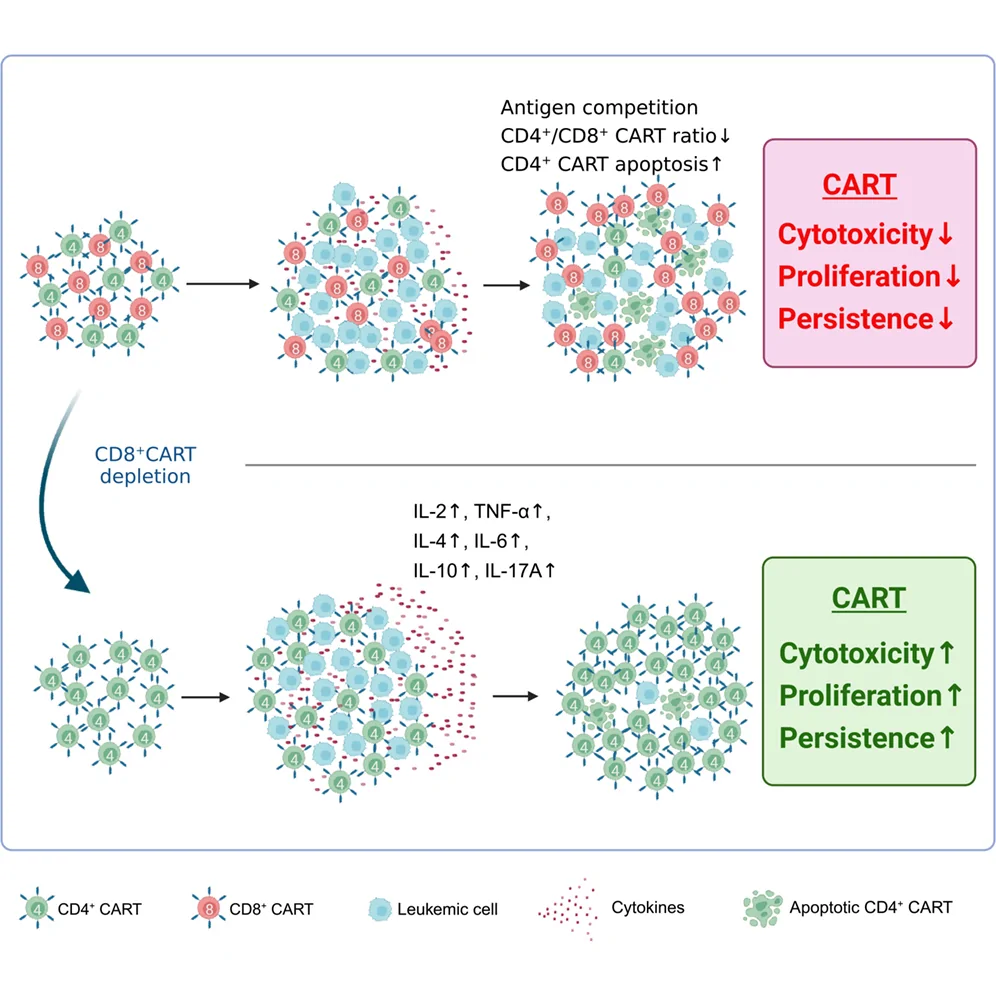

## Teaser

Sauer and colleagues report that while CART therapy is effective in hematologic malignancies, treatment failures persist. They demonstrate that pure CD4^+^ CARTs possess superior anti-tumor activity and proliferative capacity versus CD8^+^-containing products. Mechanistically, CD8^+^ CARTs impair CD4^+^ functionality via antigen competition, a previously unrecognized limitation to therapeutic efficacy.

## Introduction

The adoptive transfer of chimeric antigen receptor (CAR)-modified T lymphocytes (CARTs) has emerged as an innovative cancer immunotherapy, offering an effective treatment strategy for various malignancies. CARTs targeting CD19 and BCMA have proven to be effective for the treatment of CD19-expressing malignancies such as acute lymphoblastic leukemia, B cell non-Hodgkin’s lymphoma, and multiple myeloma. This success has led to the approval of multiple CD19- and BCMA-specific CART products by the US Food and Drug Administration and the European Medicines Agency.^1^ While CARTs can induce long-lasting remissions in many patients, primary resistance and disease relapse remain major challenges. Insufficient expansion and persistence of CARTs are frequently observed in patients with primary and secondary treatment failure to CART cell treatment, highlighting an urgent need for strategies to overcome these major limitations.^2^^,^^3^^,^^4^^,^^5^^,^^6^

Optimizing the composition of CART products with respect to content of CD4+ and CD8+ CARTs has been one approach to achieve prolonged anti-tumor activity of CARTs.^7^ So far, most clinically used CART products contain T cells with a donor-specific CD4/CD8 ratio following leukapheresis, resulting in a heterogeneous mixture of these two distinct subsets.^4^^,^^5^^,^^6^^,^^8^^,^^9^^,^^10^^,^^11^ These subsets differ significantly in their activation and exhaustion states, differentiation potential, proliferative capacity, and cytokine release profiles.^12^^,^^13^^,^^14^^,^^15^ To date, only lisocabtagene maraleucel (Liso-cel), a CD19-targeting CART product approved for the treatment of relapsed/refractory large B cell non-Hodgkin lymphoma, contains CD4^+^ and CD8^+^ central memory T (T_CM_) cells at a predefined ratio of 1:1. While Liso-cel exhibits a superior safety profile with a lower incidence of toxicities, its efficacy data are comparable to those of non-adjusted commercial products.^16^ Traditionally, CD4^+^ T cells have been considered to be “helper” cells, orchestrating the immune response by releasing cytokines and supporting other immune cells, while CD8^+^ T cells are mainly responsible for direct cytotoxic effects, targeting and destroying infected or malignant cells.^17^^,^^18^ However, emerging studies have demonstrated that CD4^+^ CART cells can directly kill tumor cells in an MHC-independent manner through death receptor-mediated cytolysis, granule exocytosis, and proinflammatory cytokines such as IFN-γ and TNF-α.^12^^,^^13^^,^^14^^,^^15^^,^^19^^,^^20^^,^^21^^,^^22^^,^^23^ Interestingly, a recent study reported that CD4^+^ CARTs were the most abundant subpopulation in the blood of two patients about 10 years after CD19-CART infusion to treat chronic lymphocytic leukemia (CLL).^24^ These results suggest that CD4^+^ CARTs may play an indispensable role as both “helpers” and “fighters.”

In this study, we sought to elucidate the significance of CD4^+^ CARTs and investigate the crosstalk between CD4^+^ and CD8^+^ subsets within CART products. Our goal was to determine the optimal CD4^+^/CD8^+^ CART cell ratio that enhances the CART functionality against acute leukemias, potentially improving therapeutic outcomes.

## Results

### Healthy-donor-derived CART products with superior functionality contain a higher percentage of CD4^+^ CARTs

**Figure 1:**
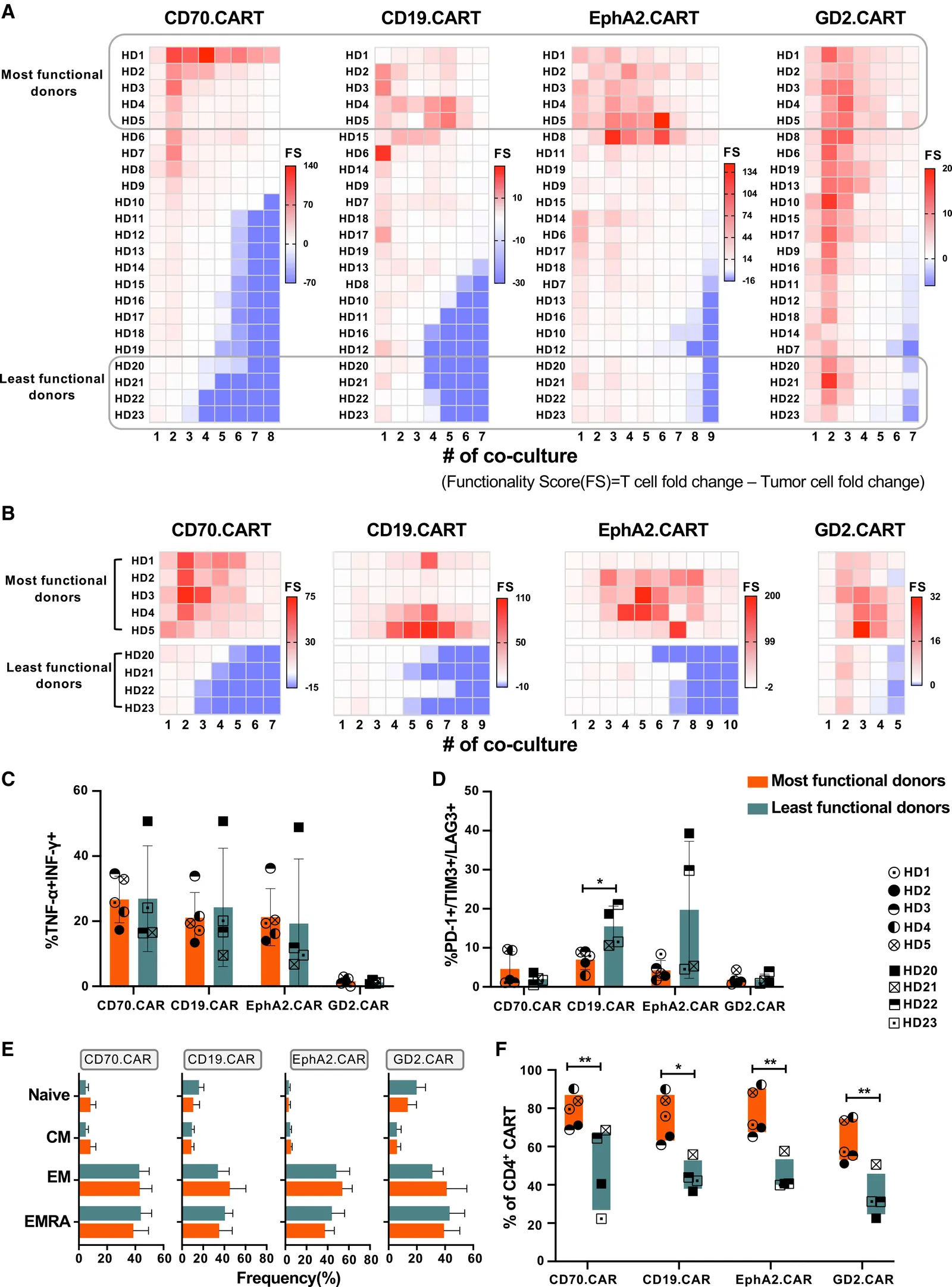
Comparative analysis of CART products derived from most potent effectors and least potent effectors (A and B) Serial co-culture assay with healthy-donor-derived CD70.CART, CD19.CART, EphA2.CART, or GD2.CART products and their respective target cells. The absolute tumor and T cell numbers at the end of each co-culture were determined by flow cytometry using CountBright counting beads. Double-gradient heatmaps depict the functionality score (FS) for each co-culture. The FS is a composite metric designed to integrate CART cell proliferation and tumor cell expansion into a single numerical value. Specifically, it is calculated by subtracting the fold change in tumor cells from the fold change in CART cells, with both values determined relative to their initial counts (red, high FS; white, FS = 0; blue, low FS). A high, positive FS indicates effective CART cell proliferation and tumor cell reduction, while a low or negative FS indicates inadequate CART cell proliferation, uncontrolled tumor growth, or both. (A) CART products were generated from 23 healthy donors (designated HD1 to HD23). Donors were stratified post hoc based on their calculated FSs across the four experimental CART products (i.e., CD70.CART, CD19.CART, EphA2.CART, GD2.CART). The donors who consistently achieved the highest FS across all tested constructs were defined as “Most functional donors” (HD1–5), while the donors who exhibited the lowest FS across the four CART products were defined as “least functional donors” (HD20–23). CD70.CARTs and Molm-13 cells were co-cultured at a 1:4 E:T ratio, CD19.CARTs and Raji cells at a 1:1 E:T ratio, EphA2.CARTs and A-549 cells at a 1:1 E:T ratio, and GD2.CARTs and LAN-1 cells at a 2:1 E:T ratio. Fresh target cells were added every 5 days for the CD70.CART, CD19.CART, and EphA2.CART and every 3 days for the GD2.CART experiments. (B) CART products from the 5 most and 4 least potent donors were co-cultured at more challenging ET ratios of 1:6 (CD70.CART), 1:2 (CD19.CART), 1:4 (EphA2.CART), and 1:1 (GD2.CART), and fresh target cells were added every 5 (CD70.CART), 3 (CD19.CART and GD2.CART), and 4 days (EphA2.CART). (C) Percentage of CART cells producing TNF-α^+^ and IFN-γ^+^ for the most and least potent effectors after 4 h of antigen stimulation at an E:T ratio of 1:1 as determined by intracellular cytokine staining followed by flow cytometry. (D) Expression of PD-1, TIM-3, and LAG-3, markers associated with T cell exhaustion, on CARTs of most and least potent effectors as determined by flow cytometry on day 10 (CD70.CARTs), day 12 (CD19.CARTs and GD2.CARTs), and day 20 (EphA2.CARTs) after co-culture assay. (E and F) Immunophenotypic characterization of CART cells between most and least potent effectors by flow cytometry on day 16 post transduction (naive: CD45RA + CCR7+; central memory [CM]: CD45RA–CCR7+; effector memory [EM]: CD45RA−CCR7−; EM T cells re-express CD45RA [EMRA]: CD45RA + CCR7−). A two-tailed *t* test was used for statistical analysis. The data represent the mean ± standard deviation (SD). ∗*p* < 0.05; ∗∗*p* < 0.01.

To investigate donor-associated factors that potentially influence the functionality of CART products, we generated CART products, targeting CD70, CD19, EphA2, and GD2 (i.e., CD70.CART, CD19.CART, EphA2.CART, GD2.CART), from activated non-selected T cells of 23 healthy donors (HD1–HD23). We assessed the *in vitro* cytotoxic and proliferative capacity of all 92 CART products by repetitive stimulation with tumor cells expressing their respective target antigen in a serial co-culture assay (Figures S1A–S1C). Based on these results, a functionality score (FS) for each donor and CAR construct was calculated, and donors were subsequently ranked according to the result of their respective FS (Figure 1A). A subsequent co-culture assay at a lower and thus more challenging effector-to-target (E:T) cell ratio confirmed the FS-based functional ranking of donors (Figure 1B).

Next, we compared the secretion of TNF-α^+^ and IFN-γ^+^ upon antigen stimulation between CART products generated from donors with high and low FS and found no statistically significant difference (Figure 1C). Furthermore, we determined the expression of proteins associated with T cell exhaustion on the surface of CARTs. While the percentage of PD-1-, TIM-3-, and LAG-3- (PD-1+/TIM-3+/LAG-3+) expressing cells was significantly higher only in CD19.CARTs with low FS, there was no difference for CD70.CARTs, EphA2.CARTs, and GD2.CARTs (Figure 1D). Subsequently, characterization of CART products revealed no significant differences in T cell differentiation (Figure 1E). However, CART products with high FS did contain a significantly increased percentage of CD4^+^ CARTs compared with those with impaired functionality (Figure 1F), suggesting that CD4^+^ CART prevalence is associated with a high FS in healthy-donor-derived CART cells.

### Increasing CD4^+^/CD8^+^ CART ratio is associated with enhanced anti-leukemic activity *in vitro*

**Figure 2:**
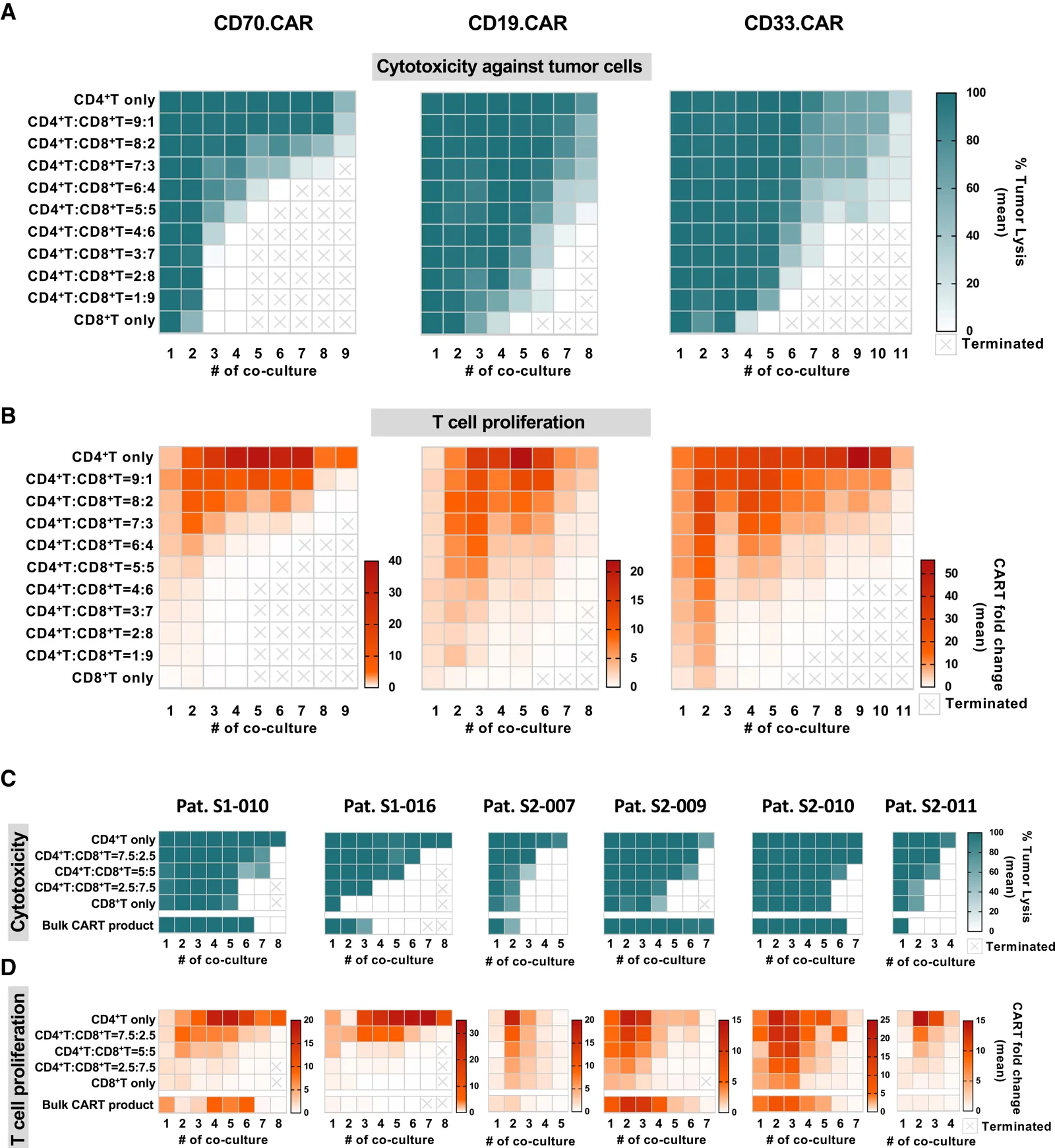
Higher CD4^+^ CART composition leads to improved anti-tumor efficacy and enhanced T cell proliferation (A and B) Serial co-culture assay with CD70.CARTs and Molm-13, CD19.CARTs and Nalm-6 and CD33.CARTs and HL-60 at an E:T ratio of 1:3 cells from 6 healthy donors. Fresh tumor cells were added every 3 (CD19.CARTs and CD33.CARTs) or every 4 days (CD70.CARTs). The absolute tumor and T cell numbers at the end of each co-culture were determined by flow cytometry using CountBright counting beads. (A) Double-gradient heatmap of tumor cell killing (blue, 100% target cell lysis; white, 0% target cell lysis). (B) Double-gradient heatmap of T cell proliferation (red, highest proliferation value; orange, baseline proliferation value; white, no proliferation). (C and D) CD19.CARTs derived from 6 patients (Pat) treated within the HD-CAR1 clinical trial were isolated using fluorescence-activated cell sorting (FACS), subsequently reconstituted at the indicated CD4/CD8 ratios, and co-cultured with Nalm-6 cells at a 1:3 E:T ratio. The same number of fresh tumor cells as in the first co-culture round was added to each well every 3 days. The absolute tumor and T cell numbers at the end of each co-culture were determined by flow cytometry using CountBright counting beads. Bulk CART product indicates the CD4^+^/CD8^+^ ratio of the transfused CART product, which is provided in Table S1. (C) Double-gradient heatmap of tumor cell killing (blue, 100% target cell lysis; white, 0% target cell lysis). (D) Double-gradient heatmap of T cell proliferation (red, highest proliferation value; orange, baseline proliferation value; white, no proliferation).

Having established that healthy-donor-derived CART products with high FS contained a significantly increased percentage of CD4^+^ CARTs, we next sought to determine the impact of the CD4/CD8 ratio on the phenotype and functionality of CART targeting AML-specific CD33 and CD70, as well as B cell malignancy-specific target CD19 (Figures S1A and S1B; Figure 2).

CD4^+^ and CD8^+^ CART subsets did not show significant differences in the frequency of CAR-expressing T cells or intensity of CAR expression as determined by the integrated mean fluorescence intensity (Figures S3A and S3B). On day 16 of CART manufacturing, CD4^+^ CART cells predominantly displayed a memory phenotype (central memory [T_CM_] and effector memory [T_EM_] T cells) as determined by the expression of CD45RA and CCR7 using flow cytometry, whereas the CD8^+^ CART subsets consisted predominantly of terminally differentiated T cells (T_EMRA_) (Figures S3C and S3D). Upon stimulation with target antigen-expressing tumor cells, the CD107a expression was significantly increased in CD8^+^ CARTs, indicating a stronger degree of degranulation (Figure S3E). In contrast, the secretion of TNF-α was significantly higher in CD4^+^ CARTs, while IFN-γ secretion was elevated for CD19- and CD33-expressing CD8^+^ CARTs but decreased for CD8^+^ CD70.CARTs (Figure S3F).

To evaluate the short-term cytotoxic potential of CD4^+^ and CD8^+^ CARTs, we stimulated the cells with target antigen-positive tumor cells and found a trend toward enhanced tumor cell killing for CD8^+^ CD70.CARTs and CD19.CARTs and even a significant advantage for the CD8^+^ CD33.CARTs (Figure S4A). Next, we sought to evaluate the impact of the CD4/CD8 composition of CARTs products on the cytolytic and proliferative capacity upon repeated antigen stimulation. We used CD4^+^ and CD8^+^ CARTs that were separated by fluorescence-activated cell sorting (FACS) to generate CART products consisting of CD4/CD8 ratios ranging between 9:1 and 1:9 as well as pure CD4^+^ and CD8^+^ CART products and repeatedly stimulated these products with target antigen-expressing tumor cells (Figures S4B and S4C). Cytotoxicity and proliferation of all CART products improved continuously with increasing CD4/CD8 ratios, and pure CD4^+^ CARTs exhibited superior functionality, while pure CD8^+^ CARTs showed the lowest proliferation and anti-tumor activity (Figures 2A and 2B). To exclude a potential association between the functional superiority of CD4^+^ CARTs and NF-κB activation mediated by the 4-1BB or CD27 co-stimulatory domains, we performed additional experiments using various second-generation constructs. These included a CLL-1-directed CAR with a CD28 domain and two FMC63-based CD19-specific CARs containing either CD28 or 4-1BB (Figures S5A and S5B), and we found a consistent association between the CD4/CD8 ratio and the functionality of the CART product (Figures S5C–S5E). Additional experiments were conducted using both the CD19- and CD70-specific CART products undergoing a significantly shortened production cycle. These products were sorted 5 days after activation and displayed a less differentiated (higher content of T_SCM_ and T_CM_ populations) in both CD4^+^ and CD8^+^ CARTs. The experiments delivered similar results as were observed with the full-length production protocol (Figures S6A–S6C).

Having demonstrated the enhanced anti-tumor activity of CD4^+^ CARTs derived from healthy donors, we asked the question of whether depletion of CD8^+^ CARTs may also enhance the functionality of patient-derived CARTs. To this end, cryopreserved third-generation CD19.CARTs derived from 6 patients with B cell malignancies who were treated within the HD-CAR1 clinical trial (NCT03676504) were thawed, and CD4^+^ and CD8^+^ CARTs were separated by FACS and repeatedly stimulated with Nalm-6 tumor cells at different CD4/CD8 ratios. Documented clinical responses in these patients ranged from complete remissions to progressive disease (Table S1). In 5 out of 6 patients, *in vitro* cytotoxicity and proliferation of CD19.CARTs could be enhanced by increasing the percentage of CD4^+^ CARTs within the product, and similar to our results with healthy donors, we observed superior proliferation and anti-tumor efficacy for pure CD4^+^ CART products compared with the original non-modified CART products, suggesting that depleting CD8^+^ CARTs to obtain a pure CD4^+^ CART product may also significantly enhance the functionality of patient-derived CART products (Figures 2C and 2D).

### CD4^+^ CART cells exhibit superior anti-tumor activity and proliferative capacity *in vivo*

**Figure 3:**
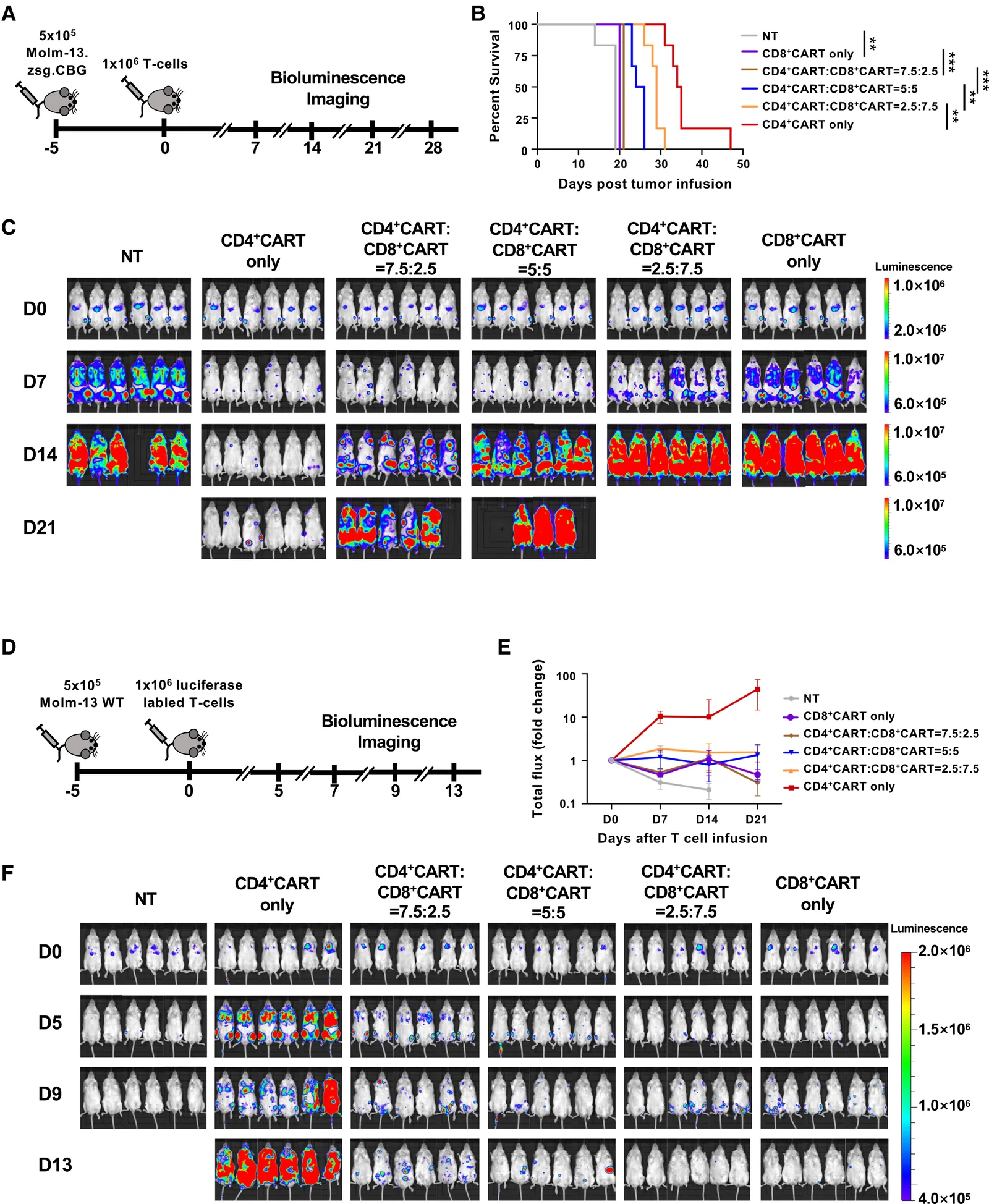
CD4^+^ CD70.CARTs have higher anti-tumor and proliferative activity *in vivo* (A) Schematic representation of the experimental setup of the Molm-13 xenograft model. 1 × 10^6^ non-transduced (NT) T cells or CD70.CART cells at various CD4^+^/CD8^+^ CART ratios were administered i.v. 5 days after i.v. injection of 5 × 10^5^ Molm-13 cells expressing click beetle green (CBG) luciferase. Bioluminescence imaging (BLI) was performed prior to T cell injection on day 0 to confirm tumor cell engraftment and continued weekly thereafter (*N* = 6 for each treatment group). (B) Kaplan-Meier survival graph of mice treated with either NT T cells or CD70.CARTs at various CD4^+^/CD8^+^ CART ratios. The statistical analysis of survival between the treatment groups was performed using the log rank (Mantel-Cox) test (*n* = 6, ∗∗*p* < 0.01; ∗∗∗*p* < 0.001). (C) Images depicting the BLI signal derived from Molm-13 cells on the specified days in mice treated with either NT T cells or CD70.CARTs at various CD4^+^/CD8^+^ CART ratios. (D) Schematic representation of *in vivo* T cell persistence experiment. NSG mice were injected with 5 × 10^5^ Molm-13 cells 5 days prior to a single dose of 1 × 10^6^ NT T cells or CD70.CARTs at various CD4^+^/CD8^+^ CART ratios also expressing CBG luciferase. T cell trafficking and proliferation were determined by BLI on days 0, 4, 9, and 13 of T cell injection (*n* = 6 for each group). (E) Fold change of total flux for each group treated with luciferase-labeled NTs or CD70.CARTs. The data represent the mean ± SD obtained from 6 mice per treatment group. (F) Images depicting the BLI signal derived from T cells on the specified days in mice treated with either NTs or CD70.CARTs at various CD4^+^/CD8^+^ CART ratios.

Next, we investigated the functionality of CD70- and CD19-directed CART products at various CD4/CD8 ratios as pure CD4^+^ and CD8^+^ CARTs *in vivo* using a murine NSG xenograft model. To this end, NSG mice were intravenously (i.v.) injected either with 5 × 10^5^ CD70-expressing Molm-13 cells genetically modified to express the click beetle green (CBG) luciferase or 1 × 10^5^ CD19-postive Nalm-6 cells expressing firefly luciferase on day 0. On day 5, after tumor cell engraftment, mice were infused i.v. with a single dose of 1 × 10^6^ CD70.CART or CD19.CART cells composed of either pure CD4^+^ or CD8^+^ CART subsets or a mixture of CD4^+^ and CD8^+^ at ratios of 3:1, 1:1, and 1:3. Animals treated with non-CAR-transduced (NT) T cells served as controls. Tumor growth was observed by weekly bioluminescence imaging (Figures 3A; S7A). Intriguingly, for both the CD70.CART and the CD19.CART model, the pure CD4^+^ CART cell products exhibited the strongest anti-leukemic activity in all treated animals compared with the CART cell products containing CD4^+^ and CD8^+^ or the pure CD8^+^ CART cells, leading to a statistically significant survival benefit. Importantly, the efficacy of CARTs gradually decreased by reducing CD4^+^/CD8^+^ T cell ratios with the pure CD8^+^ CART product showing only a marginally better tumor cell control than NT cells. (Figures 3B and 3C; S7B–S7E). The administered CART infusions were well tolerated, with no considerable weight loss observed in any of the mice (Figures S7F–S7G).

To investigate a potential correlation between the enhanced anti-tumor activity of CD4^+^ CARTs and their *in vivo* proliferation and persistence, we used our Molm-13 xenograft model with unmodified tumor cells and CD70.CARTs that were genetically modified to express a CBG luciferase fusion protein (Figure 3D). We observed a significant proliferative advantage of pure CD4^+^ CARTs over CART products that contained CD8^+^ CARTs, and analogously to the tumor cell elimination, T cell proliferation and persistence gradually decreased with increasing content of CD8^+^ CARTs. The least CART proliferation was observed in mice treated with pure CD8^+^ CARTs (Figures 3E and 3F).

### The CD4^+^ CART secretome significantly enhances the functionality of CARTs

**Figure 4:**
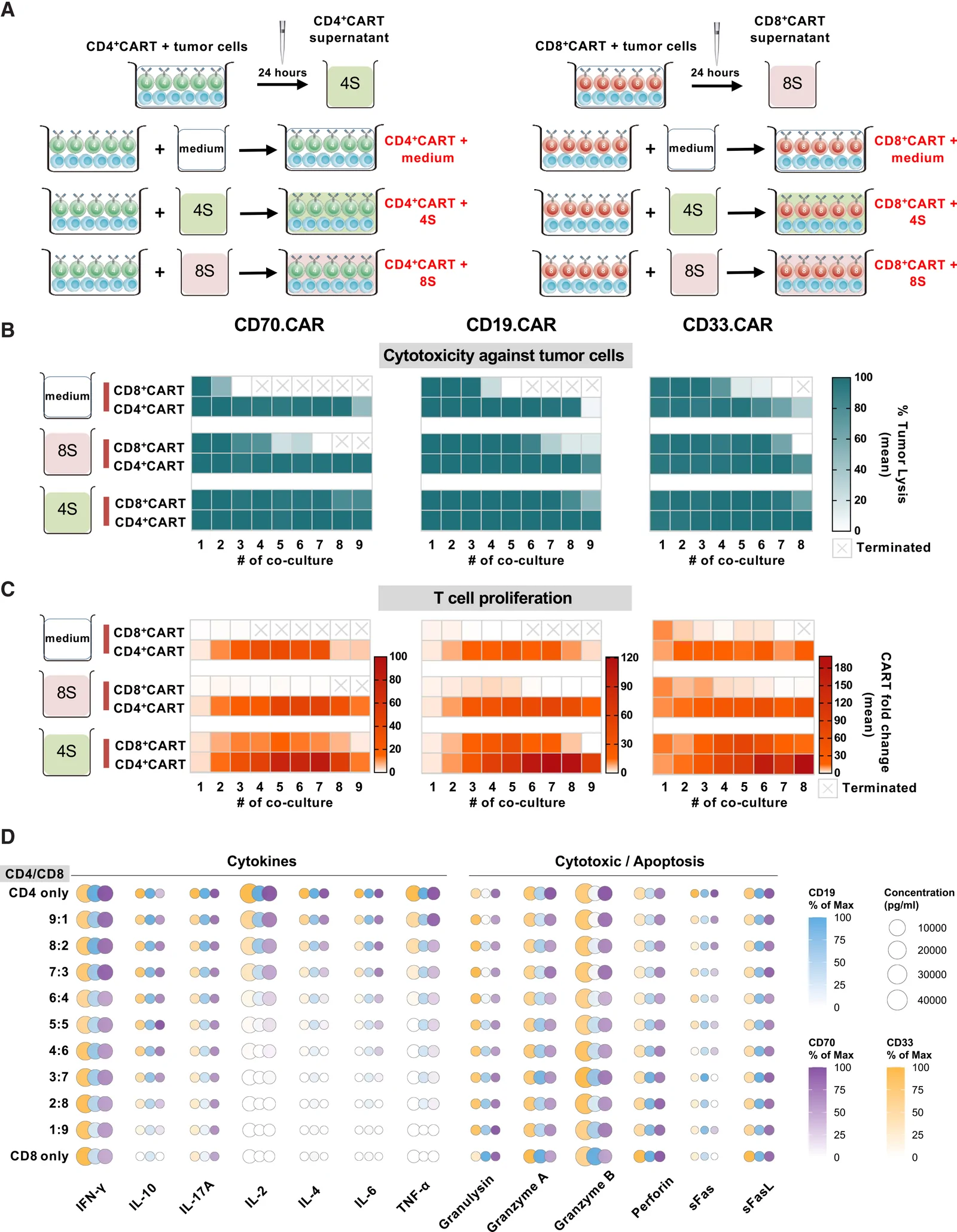
Functional impact of secretome derived from CD4^+^ CARTs and CD8^+^ CARTs (A) Schematic outline of the experimental setup. Supernatant harvested from co-cultures of CD4^+^ CARTs or CD8^+^ CARTs with appropriate target cells after 24 h is indicated as 4S or 8S. (B and C) Serial co-culture assays with CD4^+^ or CD8^+^ CART cells from 6 healthy donors and target tumor cells in the presence of 4S, 8S, or fresh medium as control. CD70.CARTs and Molm-13, CD19.CARTs and Nalm-6, and CD33.CARTs and HL-60 were co-cultured at an E:T ratio of 1:3. Fresh tumor cells were added every 3 (CD19.CARTs and CD33.CARTs) or every 4 days (CD70.CARTs). The absolute tumor and T cell numbers at the end of each co-culture were determined by flow cytometry using CountBright counting beads. (B) Double-gradient heatmap of tumor cell killing (blue, 100% target cell lysis; white, 0% target cell lysis). (C) Double-gradient heatmap of T cell proliferation (red, highest proliferation value; orange, baseline proliferation value; white, no proliferation). (D) Multiplex analysis of cytokine and cytotoxic mediator secretion by CART products at varying CD4^+^/CD8^+^ ratios. CD70.CART, CD19.CART, and CD33.CART cells were co-cultured with their respective target cells (Molm-13, Nalm-6, and HL-60) at an E:T ratio of 1:3 for 24 h. Supernatants were analyzed by bead-based multiplex immunoassay (LEGENDplex). For each analyte, three dots represent the three CAR constructs (CD33, left; CD19, center; CD70, right). Dot size indicates absolute analyte concentration (pg/mL). Color intensity reflects the percentage of maximum concentration observed within each CAR construct and analyte combination. Data represent mean values from *n* = 6 independent healthy donors per CAR construct.

To further elucidate the mechanism behind the superior efficacy of CD4^+^ CART cells, we sought to determine whether soluble factors secreted by CD4^+^ CART cells may be pivotal for their enhanced functionality. CD4^+^ and CD8^+^ CARTs were stimulated separately with their corresponding target tumor cells and the supernatant from these co-cultures were harvested 24 h after stimulation. Subsequently, a second co-culture was set up in which CD4^+^ or CD8^+^ CARTs were stimulated with target tumor cells either in the presence of supernatant harvested from CD4^+^ CART co-culture (4S), CD8^+^ CART co-culture (8S), or in the presence of fresh medium without any cytokine substitution (M). 4S, 8S, and M were freshly added with every antigen stimulation (Figure 4A). As expected, we observed improved cytotoxicity and proliferation for CD4^+^ and CD8^+^ CARTs in the presence of the 8S supernatant compared with medium control. Notably, the presence of 4S leads to even further enhanced cytotoxic and proliferative capacity of CD4^+^ and CD8^+^ CARTs (Figures 4B and 4C).

To further characterize the soluble factors secreted by CD4^+^ and CD8^+^ CARTs, we measured the supernatant concentration of several cytokines secreted by CD4^+^ and CD8^+^ CARTs when they mixed in various ratios in the co-culture 24 h after stimulation using a flow cytometry-based multiplex immunoassay. With increasing CD4/CD8 ratios, CARTs secreted higher levels of Th1-, Th2-, and Th17-related cytokines compared with products containing a higher percentage of CD8^+^ CARTs, namely IL-2, TNF-α, IL-4, IL-6, and IL-17. Additionally, CD19.CART and CD33.CART products with increased CD4/CD8 ratio also led to higher IL-10 secretion (Figure 4D).

### Direct cell-to-cell interaction with CD8^+^ CART cells impairs the functionality of CD4^+^ CART cells

**Figure 5:**
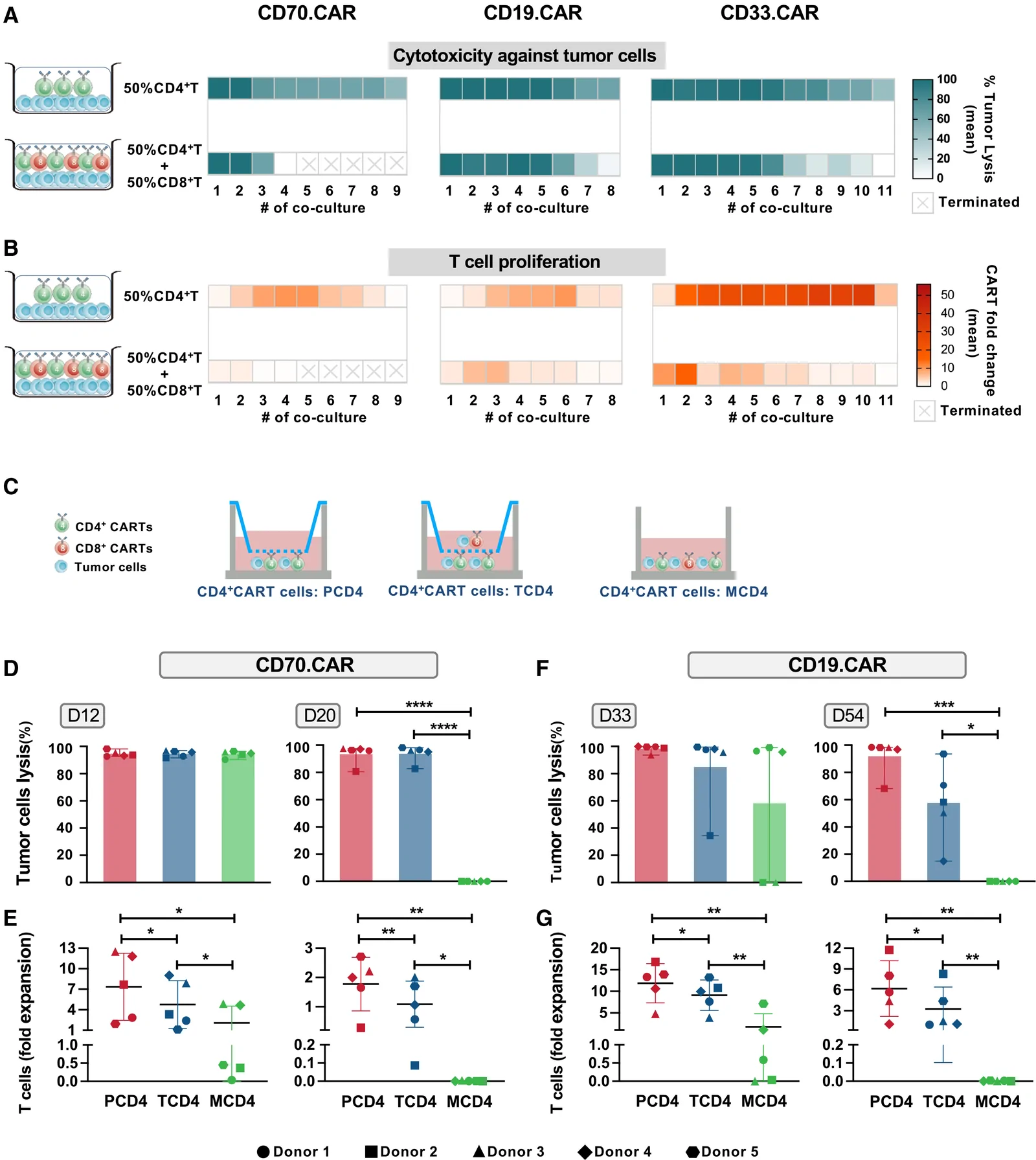
Interaction with CD8^+^ CARTs impairs the functionality of CD4^+^ CARTs (A and B) Serial co-culture assay of CD4^+^ CD70.CARTs, CD19.CARTs, and CD33.CARTs from 6 healthy donors with appropriate target cells in the absence or presence of an equal amount of CD8^+^ CARTs resulting in a 2-fold increase of the total CART number compared with the pure CD4^+^ CART conditions. Thus, E:T ratio was 1:3 for CD4^+^/CD8^+^ and 1:6 for the pure CD4^+^ CART conditions. Fresh tumor cells were added every 3 (CD19.CARTs and CD33.CARTs) or every 4 days (CD70.CARTs). The absolute tumor and T cell numbers at the end of each co-culture were determined by flow cytometry using CountBright counting beads. (A) Double-gradient heatmap of tumor cell killing (blue, 100% target cell lysis; white, 0% target cell lysis). (B) Double-gradient heatmap of T cell proliferation (red, highest proliferation value; orange, baseline proliferation value; white, no proliferation). (C) Experimental setup of the serial co-culture assay in the Transwell cell-culture device. CD70.CARTs and CD19.CARTs were co-cultured with Molm-13 or Nalm-6 cells at an E:T ratio of 1:1 and fresh tumor cells were added every 3 (CD19.CARTs) or every 4 days (CD70.CARTs) to both the top and bottom chambers. A fixed number of cells was added each time to maintain the initial predefined E:T ratio. Once the cells expanded beyond the spatial capacity in both chambers, they were transferred to larger inserts and wells to accommodate the increased volume. CD4^+^ CARTs (in the lower chamber) separated from CD8^+^ CARTs by the semipermeable membrane are indicated as TCD4, CD4^+^ CARTs cultured in the lower chamber without any CD8^+^ CARTs are indicated as PCD4, and CD4^+^ CARTs that were co-culture in the presence of CD8^+^ CARTs are indicated as MCD4. The absolute tumor and T cell numbers at the end of each co-culture were determined by flow cytometry using CountBright counting beads. The T cell proliferation was calculated as the cumulative fold expansion based on the initial cell count. This setup applies to (D)–(G). (D) Tumor cell killing and (E) T cell proliferation of CD70.CARTs on day 12 and day 20 of co-culture. The T cell proliferation was calculated as the cumulative fold expansion based on the initial cell count. (F) Tumor cell killing and (G) T cell proliferation of CD19.CARTs on day 33 and day 54 of co-culture. (*n* = 5, paired *t* test, data represent mean ± SD, ∗*p* < 0.05; ∗∗*p* < 0.01; ∗∗∗∗*p* < 0.0001).

While a mixture of CD4^+^ and CD8^+^ CART cells exhibited improved cytotoxic activity compared with pure CD8^+^ CART product, they were less efficacious in tumor cell elimination compared with pure CD4^+^ CART product. This finding prompted us to further investigate the impact of CD8^+^ CARTs on CD4^+^ CARTs. Utilizing our co-culture assay, we compared the anti-tumor efficacy and proliferation of CARTs at a CD4/CD8 ratio of 1:1 with pure CD4^+^ CART products that contained only half the amount of total CARTs. Despite this hypothetical disadvantage, the pure CD4^+^ CART product demonstrated superior cytotoxicity and proliferation compared with the product containing both CD8^+^ and CD4^+^ CART cells, suggesting that CD8^+^ cells diminished the functionality of CD4^+^ CARTs (Figures 5A and 5B; S9). However, when examining the exhaustion phenotype of CD4^+^ and CD8^+^ CART at different ratio compositions during repetitive antigen stimulation, the expression of inhibitory receptors (PD-1+/TIM-3+/LAG-3+) between these two subsets did not differ significantly (Figure S10).

To elucidate the lack of synergistic effect observed after mixing CD4^+^ and CD8^+^ CART cells, we postulated that the CD4/CD8 ratio of the CARTs within the mixture might have undergone a shift during repeated stimulation. Indeed, the composition of the cells was analyzed during the co-culture assays, which revealed an increase in the percentage of CD8^+^ T cells along with a decrease in CD4^+^ T cells (Figure S11). Specifically, this alteration in the CD4/CD8 ratio was attributable, at least in part, to the greater proliferation of CD8^+^ CART cells, which were tracked using the intracellular fluorescent label carboxyfluorescein diacetate succinimidyl ester (CFSE). The proliferation of CD8^+^ CART cells was augmented in the presence of CD4^+^ CARTs, while the expansion of CD4^+^ CARTs was diminished when they were co-applied with CD8^+^ T cells (Figure S12).

Next, to further investigate whether the inhibitory effect of CD8^+^ CARTs on CD4^+^ CARTs may be attributed to competitive consumption of cytokines, or rather direct cell-to-cell interaction, we utilized a cell-culture device with two chambers separated by a semipermeable membrane that inhibits a physical cell-to-cell contact but allows cytokines and other soluble factors to traffic. CD19- and CD70-CD4^+^ CARTs were stimulated with target cells in the bottom chamber (TCD4) while CD8^+^ CARTs were co-cultured with tumor cells in the upper chamber (Figure 5C) or vice versa (Figure S13A). Pure CD4^+^ CARTs (PCD4) or a mixture of CD8^+^ and CD4^+^ CARTs (MCD4) co-cultured with target cells served as controls (Figure 5C; S13A). In line with our previous results, PCD4 demonstrated superior anti-tumor efficacy and proliferation while the presence of CD8^+^ CARTs without direct cell-to-cell contact impaired the proliferative capacity but not the cytotoxicity of CD4^+^ CARTs at later stages of the co-culture assays (D20 and D54 for the CD70.CARTs and the CD19.CARTs, respectively). Strikingly, direct physical contact with CD8^+^ CARTs significantly reduced the anti-leukemic efficacy and proliferation of CD4^+^ CARTs with repetitive antigen stimulation, suggesting that the inhibition of CD4^+^ CART functionality by CD8^+^ CARTs is a result of direct physical interaction between the two T cell subsets (Figures 5D–5G; S13B–S13E).

### CD4^+^ CART cells acquire a more proliferative and cytotoxic effector transcriptional state in the absence of CD8^+^ CART cells

**Figure 6:**
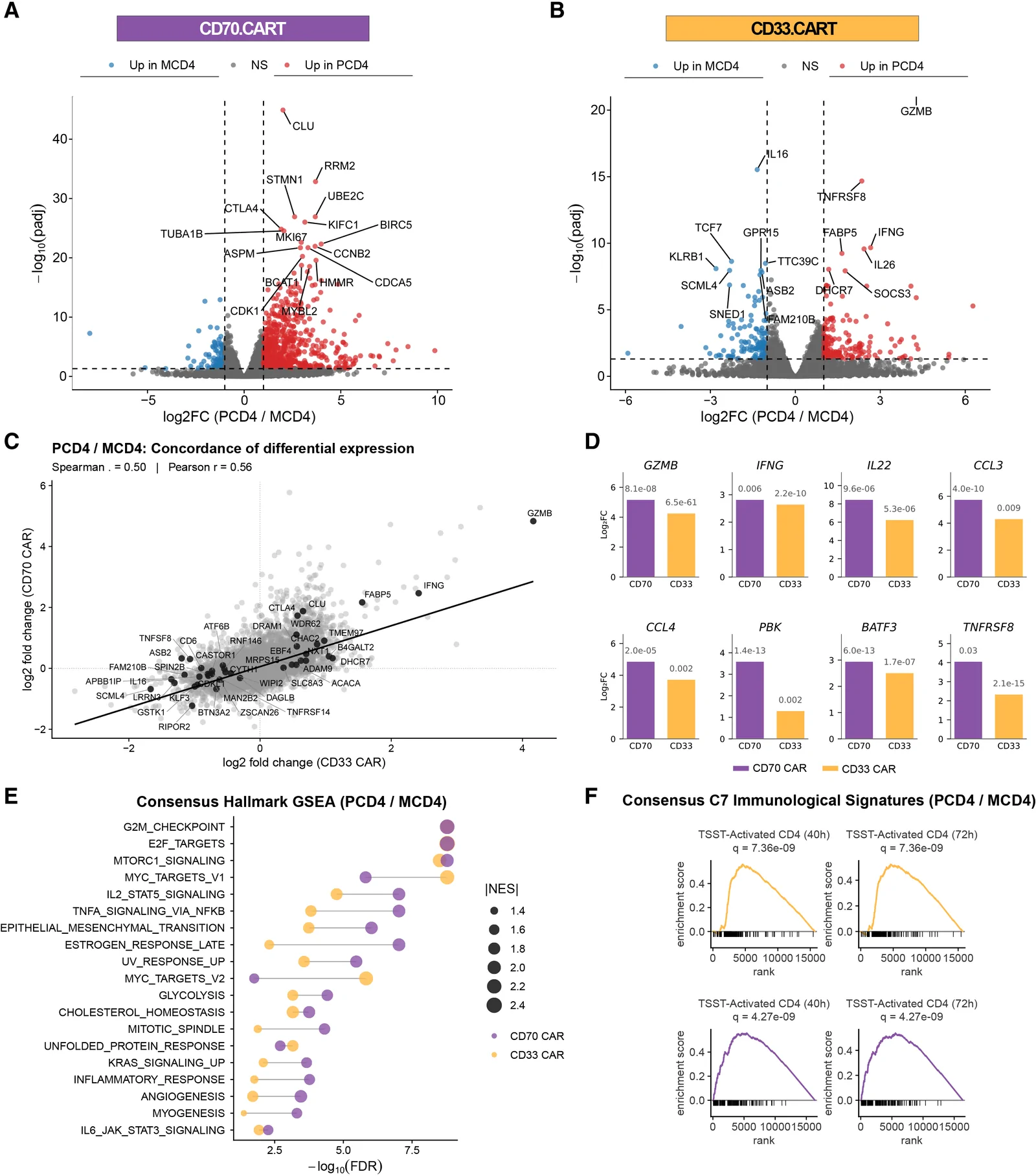
Transcriptomic profiling identifies a proliferative, cytotoxic effector polarization of CD4^+^ CARTs selectively maintained in cultures without CD8^+^ T cell intermixture Bulk RNA sequencing was performed on CD4^+^ CART cells sorted from pure CD4^+^ cultures (PCD4) or from 1:1 CD4^+^/CD8^+^ mixed cultures (MCD4) on day 12 of serial co-culture with target cells. Independent healthy donors were used for CD70.CART (*n* = 3 donors) and CD33. CART (*n* = 3 donors), representing a total of *n* = 6 unique donors across both constructs. (A and B) Volcano plots depicting differentially expressed genes between PCD4 and MCD4 for CD70.CART (*n* = 3 donors) (A) and CD33.CART (*n* = 3 donors) (B). Red dots indicate genes significantly upregulated in PCD4; blue dots indicate genes significantly upregulated in MCD4; gray dots indicate genes not meeting significance thresholds (adjusted *p* < 0.05, |log_2_FC| ≥ 1). Selected genes are labeled. (C) Concordance of differential expression between CD70.CART (*n* = 3 donors) and CD33.CART (*n* = 3 donors). Each point represents one gene; x axis shows log_2_ fold change (PCD4/MCD4) in CD33.CART cells; y axis shows log_2_ fold change in CD70.CART cells. Spearman ρ = 0.50; Pearson r = 0.56. (D) Log_2_ fold change estimates from DESeq2 for selected genes associated with cytotoxic effector function (*GZMB*), secretory activity (*IFNG, IL22*), activation state (*CCL3*, *CCL4*, *TNFRSF8*), proliferation (*PBK*), and transcriptional regulation (*BATF3*). Purple bars indicate CD70.CART (*n* = 3 donors); gold bars indicate CD33.CART (*n* = 3 donors). Bars represent DESeq2 log_2_ fold change point estimates (PCD4/MCD4), which are derived from a generalized linear model fit across all biological replicates within each CAR construct; FDR-adjusted *q* values from DESeq2 Wald tests are displayed above each bar. (E) Consensus Hallmark gene set enrichment analysis (GSEA) showing pathways significantly enriched in PCD4 cells in both CAR constructs (FDR < 0.05). Purple indicates CD70.CART (*n* = 3 donors); gold indicates CD33.CART (*n* = 3 donors). Dot size corresponds to absolute normalized enrichment score (|NES|); x axis position indicates −log_10_(FDR). (F) Gene set enrichment analysis (GSEA) of C7 immunologic signatures reveals striking enrichment of toxic shock syndrome toxin (TSST)-induced CD4^+^ memory T cell activation gene sets in PCD4 compared with MCD4 CART cells. Left two panels: CD70.CART (purple, *n* = 3 donors); right two panels: CD33.CART (gold, *n* = 3 donors). TSST 40H and TSST 72H denote gene sets derived from CD4^+^ memory T cells stimulated with TSST for 40 or 72 h, respectively. Enrichment scores and gene ranks are shown; displayed *q* values are permutation-based GSEA *q* values.

To define the transcriptional programs associated with differential CD4^+^ CART cell functionality, we performed bulk RNA sequencing on CD4^+^ CD70- and CD33-directed CART cells cultured either alone (PCD4) or together with CD8^+^ CART cells (MCD4) during repetitive antigen stimulation. Differential expression analysis revealed substantial transcriptional remodeling in PCD4 relative to MCD4 cells across both CAR constructs (Figures 6A and 6B). In CD70.CART cells, we identified 1,847 differentially expressed genes (adjusted *p* < 0.05, |log_2_FC| ≥ 1), while CD33.CART cells yielded 1,203 differentially expressed genes meeting the same thresholds. Despite using entirely different healthy donors for each CAR construct and targeting distinct tumor antigens, the transcriptional changes showed a striking degree of concordance. Comparison of log_2_ fold changes across all detected genes revealed a strong positive correlation between the two CAR specificities (Spearman ρ = 0.50, Pearson r = 0.56; Figure 6C), indicating that the transcriptional program distinguishing PCD4 from MCD4 cells is conserved and independent of CAR target or individual donor characteristics. Among the most prominently upregulated genes in PCD4 cells were those encoding cytotoxic effector molecules, most notably *GZMB* (granzyme B), which showed robust upregulation in both CD70.CART (log_2_FC = 5.2, adjusted *p* = 8.1 × 10^−8^) and CD33.CART cells (log_2_FC = 4.8, adjusted *p* < 1 × 10^−61^). Additional effector-associated transcripts including *IFNG, IL22, CCL3, CCL4*, and *TNFRSF8* were concordantly elevated in PCD4 cells across both CAR constructs (Figure 6D). The coordinated upregulation of these genes is consistent with polarization toward a cytotoxic effector-like CD4^+^ state capable of direct tumor cell elimination. To systematically interpret these transcriptional changes at the pathway level, we performed gene set enrichment analysis (GSEA) using curated Hallmark gene sets and identified pathways reaching significance (false discovery rate [FDR] < 0.05) in both CAR construct analyses (Figure 6E). PCD4 CART cells demonstrated robust enrichment of pathways associated with cell-cycle progression and proliferation, with G2M_CHECKPOINT (NES = 2.4) and E2F_TARGETS (NES = 2.3) ranking among the most highly enriched gene sets. Additional proliferation-associated pathways including MYC_TARGETS_V1, MYC_TARGETS_V2, and MITOTIC_SPINDLE were similarly enriched, reflecting the enhanced expansion capacity observed functionally in PCD4 cultures. Metabolic reprogramming was also evident, with significant enrichment of MTORC1_SIGNALING and GLYCOLYSIS pathways, consistent with the increased biosynthetic and energetic demands of actively proliferating effector cells. Furthermore, inflammatory signaling pathways including TNFA_SIGNALING_VIA_NFKB and IL2_STAT5_SIGNALING were enriched in PCD4 cells, indicating heightened activation of canonical T cell receptor and cytokine-driven transcriptional programs. Examination of immunologic gene signatures revealed an unexpected finding: pure CD4^+^ CARTs exhibit exceptionally strong enrichment of T cell activation programs associated with toxic shock syndrome toxin (TSST) exposure (Figure 6F). GSEA demonstrated highly significant enrichment of TSST-induced CD4^+^ memory T cell signatures at both 40-h and 72-h timepoints (as represented by the GSE36476_CTRL_VS_TSST_ACT_40H_MEMORY_CD4_TCELL_YOUNG_DN and GSE36476_CTRL_VS_TSST_ACT_72H_MEMORY_CD4_TCELL_YOUNG_DN annotated pathways), with this pattern observed consistently across both CD70 and CD33 CAR models.

Together, these transcriptomic data define a distinct and highly reproducible CD4^+^ CART cell state in the absence of CD8^+^ CARTs characterized by coordinated activation of proliferative, metabolic, and cytotoxic effector gene expression programs. The concordance of these findings across independent CAR constructs, donor backgrounds, and target antigens supports the conclusion that the presence of CD8^+^ CARTs fundamentally constrains the activation potential of CD4^+^ CART cells through a conserved mechanism.

### Quantitative proteomics validates the proliferative effector polarization of pure CD4^+^ CART cells and identifies interferon-associated stress signaling in mixed cultures

**Figure 7:**
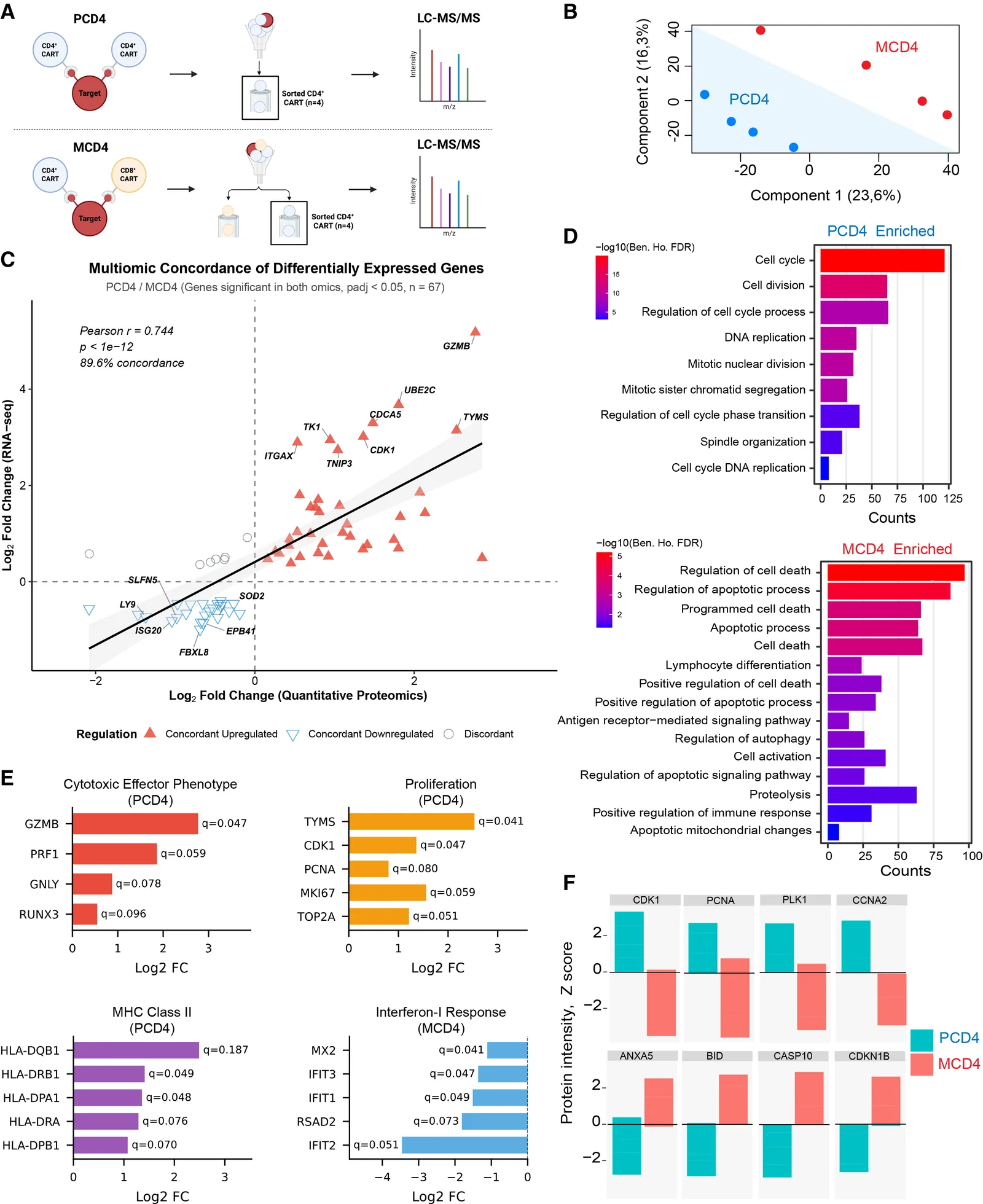
Quantitative proteomic analysis complements transcriptomic profiling and reveals interferon-associated stress signaling in CD4^+^ CARTs cultured with CD8^+^ T cell intermixture Quantitative label-free proteomic analysis was performed on CD70-specific CD4^+^ CART cells sorted from pure CD4^+^ cultures (PCD4) or from 1:1 CD4^+^/CD8^+^ mixed cultures (MCD4) on day 12 of serial co-culture with Molm-13 cells (*n* = 4 independent healthy donors). Protein quantification was performed using MaxQuant (version 1.5.5.2) with the MaxLFQ algorithm; statistical analysis was performed in Perseus (version 2.0.7.0) on log_2_-transformed LFQ intensities using permutation-based FDR correction (FDR < 0.05). (A) Schematic representation of the experimental workflow. PCD4 and MCD4 CD70.CART cells were co-cultured with Molm-13 cells at an E:T ratio of 1:3 with fresh target cells added every 4 days. On day 12, CD4^+^ CART cells were re-isolated by fluorescence-activated cell sorting (FACS) for proteomic analysis. Figure was created with Biorender.com. (B) Principal component analysis (PCA) of proteomic profiles showing separation of PCD4 (blue, *n* = 4 donors) and MCD4 (red, *n* = 4 donors) samples. (C) Multi-omic concordance analysis integrating RNA sequencing (CD70.CART, *n* = 3 donors) and quantitative proteomics (CD70.CART, *n* = 4 donors). The x axis depicts log_2_ fold change (PCD4/MCD4) from quantitative proteomics; y axis depicts log_2_ fold change from RNA-seq. Only genes meeting significance thresholds in both datasets (adjusted *p* < 0.05) are shown (*n* = 67 genes). Red triangles indicate concordant upregulation in PCD4; blue triangles indicate concordant downregulation in PCD4; gray circles indicate discordant regulation. Pearson r = 0.744, *p* < 1 × 10^−12^, 89.6% concordance. (D) Gene Ontology Biological Process (GOBP) enrichment analysis of differentially abundant proteins. Upper panel: pathways enriched in PCD4; lower panel: pathways enriched in MCD4 (magenta/purple bars). Bar color intensity corresponds to −log_10_(Benjamini-Hochberg FDR). (E) Log_2_ fold change (PCD4/MCD4) for selected proteins grouped by functional annotation: cytotoxic CD4 phenotype (GZMB, PRF1, GNLY, RUNX3), proliferation (TYMS, CDK1, PCNA, MKI67, TOP2A), MHC class II (HLA-DQB1, HLA-DRB1, HLA-DPA1, HLA-DRA, HLA-DPB1), and interferon response (MX2, IFIT3, IFIT1, RSAD2, IFIT2). Bars represent log_2_ fold change derived from comparison of PCD4 (*n* = 4 donors) versus MCD4 (*n* = 4 donors); displayed *q* values are permutation-based FDR-adjusted values from Perseus. (F) *Z* score normalized protein intensities for selected cell-cycle regulators (upper row: CDK1, PCNA, PLK1, CCNA2) and apoptosis-associated proteins (lower row: ANXA5, BID, CASP10, CDKN1B). Cyan bars indicate PCD4; red bars indicate MCD4. Each bar represents the mean *Z* score across *n* = 4 donors per condition.

To complement our transcriptomic profiling and validate these findings at the protein level, we performed label-free quantitative proteomic analysis of CD4^+^ CART cells using the same experimental framework of FACS-based subset purification followed by repetitive antigen stimulation (Figure 7A). Proteomic analyses were conducted in the CD70.CAR model using CD4^+^ CART cells derived from four independent healthy donors, with cells harvested on day 12 of co-culture to match the time point used for transcriptomic profiling. Using data-dependent acquisition mass spectrometry, we quantified 6,847 proteins at a peptide and protein FDR of 1%, providing comprehensive coverage of the CD4^+^ CART cell proteome. Proteomic profiling revealed marked differences between PCD4 and MCD4 CART cells (Figure 7B).

To assess the fidelity of transcriptional changes at the protein level, we integrated transcriptomic and proteomic datasets by comparing log_2_ fold changes for genes detected as significantly differentially expressed in both analyses. This integration revealed strong multi-omic concordance, with a Pearson correlation coefficient of r = 0.74 (*p* < 1 × 10^−12^) and 89.6% of shared features exhibiting consistent directionality across both omics layers (Figure 7C). Genes concordantly upregulated in PCD4 cells at both the transcriptional and protein level included key effector molecules (*GZMB*) and cell-cycle regulators (*CDCA5*, *TK1*, *TYMS*, *CDK1*), while concordantly downregulated features included interferon-stimulated genes (*ISG20*, *SLFN5*) and immunomodulatory receptors (*LY9*).

Pathway enrichment analysis of differentially abundant proteins confirmed the biological themes identified transcriptomically (Figure 7D). Pure CD4^+^ CARTs cells exhibited significant enrichment of Gene Ontology Biological Process terms related to cell cycle (−log_10_ FDR > 15), cell division, DNA replication, and mitotic processes. Key cell-cycle-associated proteins including CDK1, PCNA, PLK1, and CCNA2 were uniformly elevated in pure CD4^+^ CARTs (Figure 7F), consistent with the enhanced proliferative capacity observed functionally. Furthermore, cytotoxic effector-associated proteins such as granzyme B (GZMB; *q* = 0.047), perforin (PRF1; *q* = 0.059), granulysin (GNLY; q = 0.078), and the transcription factor RUNX3 (*q* = 0.096) showed significant upregulation. Proliferation-associated proteins were similarly elevated, with thymidylate synthase (TYMS; *q* = 0.041), CDK1 (*q* = 0.047), PCNA (*q* = 0.080), MKI67 (*q* = 0.059), and TOP2A (*q* = 0.051) all significantly upregulated in pure CD4^+^ CARTs (Figure 7E). Notably, pure CD4^+^ CARTs also demonstrated coordinated upregulation of proteins associated with MHC class II (Figure 7E).

Conversely, mixed CD4^+^ CARTs showed enrichment of pathways related to regulation of cell death, apoptotic processes, and programmed cell death (−log_10_ FDR > 4), aligning with our prior observation of increased apoptosis in CD4^+^ CART cells cultured in the presence of CD8^+^ T cells (Figures S14A–S14E). Among the apoptosis-associated proteins enriched in mixed CD4^+^ CARTs, the pro-apoptotic BH3-only protein BID was significantly elevated, as were ANXA5, CASP10, and the cell-cycle inhibitor CDKN1B (p27) (Figure 7F).

Mixed CD4^+^ CARTs were also characterized by a pronounced type I interferon-associated protein signature with upregulated interferon-stimulated proteins including MX2 (*q* = 0.041), IFIT3 (*q* = 0.047), IFIT1 (*q* = 0.049), RSAD2 (*q* = 0.073), and IFIT2 (*q* = 0.051) (Figure 7E). Together, these proteomic data extend the transcriptomic characterization of CD4^+^ CART cell states by validating key functional programs such as proliferation, cytotoxic effector differentiation, and MHC class II induction at the protein level.

### Antigen competition between CART cell subsets dictates CD4^+^ CART cell activation dynamics and functional fate

**Figure 8:**
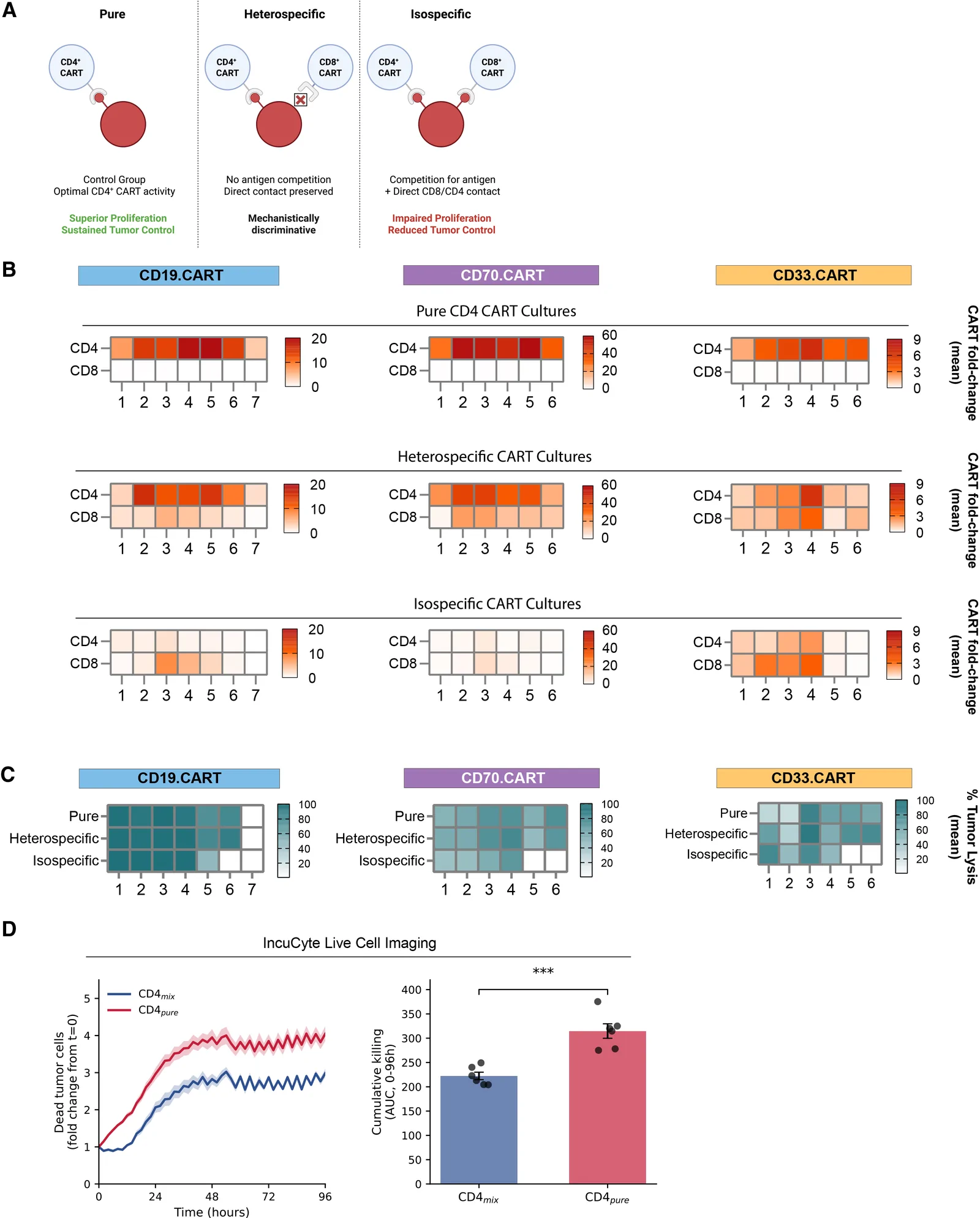
Antigen competition, rather than direct cell-cell contact, is the dominant determinant of CD4^+^ CART cell dysfunction in mixed cultures (A) Schematic representation of experimental design to discriminate antigen competition from direct CD4-CD8 cell contact. Pure: CD4^+^ CART cells cultured alone with cognate antigen-expressing tumor cells (control condition representing maximal CD4^+^ activation). Isospecific: CD4^+^ and CD8^+^ CART cells express identical CARs targeting the same tumor antigen, resulting in both antigen competition and direct cell-cell contact. Heterospecific: CD4^+^ CART cells target tumor-expressed antigen, while CD8^+^ CART cells express a CAR against an antigen absent from the tumor, thereby abolishing antigen competition while preserving physical CD4-CD8 co-localization. Heterospecific combinations: CD33.CARTs with HL-60 tumor cells (CD8^+^ cells expressing anti-CD19 CAR); CD70.CARTs with Molm-13 tumor cells (CD8^+^ cells expressing anti-CD19 CAR); CD19.CARTs with Nalm-6 tumor cells (CD8^+^ cells expressing anti-CD33 CAR). Figure was created with Biorender.com. (B) Serial co-culture assays comparing CD4^+^ and CD8^+^ CART cell proliferation under pure, heterospecific, and isospecific conditions for CD19.CART (left), CD70.CART (middle), and CD33.CART (right). Heatmaps depict mean fold expansion (*n* = 4 donors) of CD4^+^ (upper row) and CD8^+^ (lower row) CART cells at each successive co-culture round (x axis). (C) Tumor cell control under pure, heterospecific, and isospecific conditions. The absolute tumor and T cell numbers at the end of each co-culture were determined by flow cytometry using CountBright counting beads (*n* = 4). Double-gradient heatmap of tumor cell killing (blue, 100% target cell lysis; white, 0% target cell lysis); double-gradient heatmap of T cell proliferation (red, highest proliferation value; orange, baseline proliferation value; white, no proliferation). (D) Functional persistence of culture condition-induced phenotypes in CD4^+^ CART cells. CD4^+^ CART cells were re-sorted from either pure CD4^+^ cultures (PCD4, red) or from mixed CD4^+^/CD8^+^ isospecific cultures (MCD4, blue) on day 12 and then re-challenged with fresh tumor cells in the absence of CD8^+^ CART cells to assess retention of functional differences. Left panel: real-time cytotoxicity measured by IncuCyte live-cell imaging over 96 h; y axis shows fold change in dead tumor cells relative to t = 0; lines represent mean, and shaded regions represent SEM (*n* = 6 donors). Right panel: cumulative killing quantified as area under the curve (AUC, 0–96 h); each dot represents one independent healthy donor (*n* = 6); bars indicate mean ± SEM. ∗∗∗*p* < 0.001, paired two-tailed *t* test.

Previous Transwell experiments established that impairment of CD4^+^ CART cell function requires close physical association with CD8^+^ CART cells (Figures 5C–5G; S13) but could not resolve whether the dysfunctional is caused by direct intercellular signaling or competition for shared antigenic resources. To mechanistically disentangle these possibilities, we developed a dichotomous co-culture system designed to preserve CD4-CD8 proximity while selectively uncoupling antigen competition (Figure 8A). In isospecific co-cultures, CD8^+^ CART cells express an identical CAR and compete for the same tumor-associated antigen. In heterospecific co-cultures, CD8^+^ CART cells express a CAR directed against an antigen not expressed by the co-cultured tumor cells, maintaining spatial co-localization and contact-dependent interactions while eliminating competition for the target antigen. Functional analyses utilizing three different CAR constructs (CD19, CD70, and CD33) yielded similar results. CD4^+^ CARTs cultured under heterospecific conditions displayed markedly improved proliferation (Figure 8B) and tumor control (Figure 8C) compared with isospecific co-cultures, similar to pure CD4^+^ CARTs, suggesting that the antigen competition rather than the physical interaction with CD8.CARTs causes the functional impairment of CD4^+^ CARTs. Notably, CD8^+^ CART cells in heterospecific cultures lacking cognate antigen nonetheless demonstrated measurable expansion, likely reflecting bystander proliferation driven by CD4^+^-derived cytokines (Figure 8B). To validate that competitive antigen access is the primary mechanism restricting CD4^+^ CART cell function in mixed cultures, we extended the heterospecific co-culture system with a dual heterospecific arm in which CD8^+^ CART cells were provided with a second, separately expressed tumor antigen, thereby enabling productive CD8^+^ engagement while eliminating competition for the CD4^+^-targeted antigen (Figure S15A). In the anti-CD19 CART arm, cumulative CD4^+^ CART cell expansion was reduced under isospecific conditions relative to both pure CD4^+^ cultures and dual heterospecific conditions, with consistent numerical trends observed across all donors in the anti-CD33 arm (Figures S15B and S15D). CD8^+^ CART cells in dual heterospecific cultures expanded robustly upon engagement with their cognate secondary tumor target but did not suppress CD4^+^ CART cell proliferation, demonstrating that CD8^+^ expansion per se does not account for CD4^+^ dysfunction (Figures S15C and S15E). These data support the hypothesis that competitive consumption of shared tumor antigen by CD8^+^ CART cells is the principal mechanism constraining CD4^+^ CART cell function in mixed products.

To determine whether antigen competition induces durable functional impairment, we performed real-time kinetic cytotoxicity analyses using IncuCyte live-cell imaging. CD4^+^ CART cells were re-sorted from either pure or mixed co-cultures after serial stimulation and re-challenged with fresh tumor cells. Strikingly, kinetic profiling over 96 h revealed a significantly enhanced tumor cell killing capacity of pure CD4^+^ CARTs compared with CD4^+^ CARTs isolated from mixed co-cultures (mean area under the curve [AUC]: 310 versus 224; *p* < 0.001; Figure 8D), indicating that antigen competition induces lasting alterations in CD4^+^ CART cell programming rather than acute, reversible suppression.

Collectively, these data establish antigen competing CD8^+^ CART cells as the central mechanism of lasting CD4^+^ CART cell dysfunctionality in CART products containing CD4^+^ and CD8^+^ CARTs and suggest that CD4^+^ CARTs, in the absence of competition, achieve a highly activated state with enhanced proliferative and cytotoxic capacity.

## Discussion

Aiming to identify donor-specific factors associated with superior functionality of CART cells independent of structural differences within the CAR constructs themselves, we found that CART products targeting hematologic malignancies as well as solid tumors exhibited enhanced *in vitro* anti-tumor efficacy and proliferative capacity if they contained a higher percentage of CD4^+^ CART cells. *In vitro* co-culture assays and murine xenograft models confirmed the functional superiority of pure CD4^+^ CART cells compared with all products containing CD8^+^ CART cells. The elimination of CD8^+^ CART cells augmented the *in vitro* activity of CART products derived from patients treated with third-generation CD19.CART cells in an investigator-initiated trial at our institution (NCT03676504). Critically, our integrated transcriptomic, proteomic, and functional analyses identified antigen competition as a previously unrecognized mechanism limiting CD4^+^ CART cell function in mixed products, providing a unifying framework for understanding the impaired performance of CD4^+^ CART cells when co-cultured with CD8^+^ CART cells.

The optimal composition of CART products with respect to the content of CD4^+^ and CD8^+^ CARTs remains a matter of ongoing investigation, and in the majority of clinical trials, the CD4/CD8 ratio of the CART products was determined by the lymphocyte composition of the donor.^7^ For lisocabtagen maraleucel, a CD19-directed second-generation CART product containing a 4-1BB co-stimulatory, a 1:1 ratio of CD4^+^ naive (T_N_) and CD8^+^ central memory (T_CM_) T cells was demonstrated to have optimal anti-tumor activity in B cell lymphoma animal models.^14^

The superior anti-leukemic activity of pure CD4^+^ CARTs that we observed in this study was not necessarily expected as CD4^+^ T cells have been traditionally considered to function as helper cells, while CD8^+^ T cells are supposed to be the mediator of cytotoxicity.^17^^,^^25^ However, evolving evidence has demonstrated that CD4^+^ T cells can also serve as the “cytotoxic” cells and exhibit a direct tumor-cell-targeting cytotoxicity.^21^^,^^26^^,^^27^ Indeed, Both CD4^+^ and CD8^+^ CART cells are capable of forming immune synapses after engagement with the target cells, hence mediating tumor cell elimination by degranulation and ligand-based lytic pathways.^20^^,^^28^^,^^29^ Furthermore, CD4^+^ CART cells have shown superior efficacy over CD8^+^ CART cells in enhancing host immunity, which could potentially reduce the risk of antigen-negative relapse.^28^ Several other studies align with our findings demonstrating that CD4^+^ CARTs alone exhibit a superior capability for eliminating target cells.^12^^,^^13^^,^^15^^,^^19^ Agarwal et al. demonstrated that administering CD4-targeted (CD4-LV) and CD8-targeted lentiviral vector (CD8-LV) into NSG mice successfully generated murine CD4^+^ CART cells and CD8^+^ CART cells *in vivo*. Their results indicated that mice treated with CD4-LV achieved faster and more effective tumor cell elimination compared with those receiving CD8-LV alone or a combination of both vectors.^12^ In a murine orthotopic glioblastoma (GBM) model, Wang et al. showed that CD4^+^ IL13RA2 CAR T cells outperformed CD8^+^ CART cells, exhibiting sustained cytotoxicity and recursive killing potential even under repetitive tumor challenges. Furthermore, they showed the maintenance of the CD4^+^ subset positively correlated with the recursive killing ability of CART products derived from GBM patients.^15^ Both of these studies attribute the superior performance of CD4^+^ CART cells to the greater susceptibility of CD8^+^ CART cells to exhaustion. Notwithstanding, the coinhibitory-related markers (PD-1, TIM-3, LAG-3) are comparable between CD4^+^ and CD8^+^ CART cells in our co-culture experiment.

In contrast to our findings, other groups have demonstrated the superiority of CD8^+^ CARTs over CD4^+^ CARTs or at least the need for the presence of both subsets in the most potent CART products. Moeller et al. and Adusumilli et al. demonstrated that a defined 1:1 mixture of CD4^+^ and CD8^+^ CART cells confers superior and more durable anti-tumor reactivity compared with single subsets, within the context of solid tumor models of breast carcinoma and pleural mesothelioma, respectively.^19^^,^^27^ Boulch et al. demonstrated that murine CD19-targeted CD8^+^ CART cells exhibited a higher capacity to eliminate tumors as compared with CD4^+^ CART cells in an immune-replete, MYC-driven B cell lymphoma animal model.^28^^,^^30^ However, they also reported that IFN-γ production was the dominant mechanism for tumor elimination by CD4^+^ CD19.CART cells in their model, but the tumor cells were not sensitive to pro-apoptotic effect of IFN-γ.^30^ The comparability of these studies with our data is limited. The immunosuppressive and metabolically hostile tumor microenvironment of solid tumors differs fundamentally from the acute leukemia models utilized in our study. In liquid tumors, the rapid expansion and intrinsic cytotoxic potential of CD4^+^ CART cells may suffice for robust tumor clearance. Furthermore, Sommermeyer et al. compared CD19.CART products that differed not only with respect to their CD4 and CD8 expression but also contained T cell subsets of distinct differentiation status (naive, central, and effector memory and terminally differentiated), which further alters the functional characteristics of these CART products, whereas we characterized bulk CD4^+^ and CD8^+^ CART products.^14^ Bulk populations more closely reflect standard clinical manufacturing but harbor distinct intrinsic differentiation profiles. Moreover, while Lee et al. demonstrated that the presence of CD8^+^ cells is not detrimental during the early antigen-independent production phase,^31^ our data demonstrate a negative impact of the presence of CD8^+^ CART cells on the CD4^+^ CART cells during prolonged antigen exposure and tumor engagement.

Our findings raise the possibility that selective depletion of CD8^+^ CART cells from clinical products could enhance therapeutic efficacy by maximizing CD4^+^ CART cell activation potential. This approach is supported by clinical observations that CD4^+^ CART cells predominate in patients achieving decade-long remissions following CD19.CART therapy, suggesting that CD4^+^ T cells possess intrinsic features that favor long-term persistence.^24^

However, the TSST-like activation signature observed in pure CD4^+^ CART cells underscores a critical safety consideration: maximal CD4^+^ T cell activation is inherently linked to robust inflammatory cytokine production. While we did not observe any clinical signs of hyperinflammation in our murine xenograft models, the enhanced anti-leukemic activity of pure CD4^+^ CARTs *in vitro* and *in vivo* was associated with increased CART proliferation, and higher levels of Th1- and Th2-related cytokines raise the concern of more severe treatment-related toxicity. In fact, evidence suggests that higher *in vivo* expansion of CART cells correlates with increased overall response rates but also a heightened incidence of cytokine-release syndrome (CRS) in patients with B cell malignancies and multiple myeloma.^4^^,^^32^ Owing to their robust proliferative capacity and cytokine release profile, CD4^+^ CART cells may effectively stimulate myeloid cells to produce inflammatory mediators. Clinical studies have indicated associations between CRS and other factors such as disease burden, co-stimulatory domains, manufacturing procedures, and T cell differentiation status.^13^^,^^16^^,^^33^^,^^34^ Findings from two independent studies suggested that CD4^+^ CART cells may be more prone to inducing CRS,^13^^,^^35^ a major adverse event associated with CART cell therapy in B cell malignancies. One study utilized a murine-derived CD19.CART cell therapy in an immunocompetent mouse model, reporting that CD4^+^ CART cell therapy favors the occurrence of CRS in cases of high tumor burden, while at low tumor burden, no discernible toxicities were observed, and a therapeutic benefit was still noted.^35^ Another study using immunodeficient mice reconstituted with human CD34^+^ stem cells and engrafted with Nalm-6 tumor cells demonstrated superior long-term responses accompanied by CRS in pure CD4^+^ CART cells compared with pure CD8^+^ CD19-directed CART cells.^13^ However, in another research using the same humanized mouse model, both the CD4^+^ CART group and mixed CD4^+^/CD8^+^ CART group exhibited similar CRS-like symptoms in the context of an effective anti-tumor response.^12^ To date, no clinical trials have been conducted using pure CD4^+^ CART cells for treatment in real-world settings. Therefore, the induction mechanisms and pathophysiology of CRS associated with CD4^+^ CART cells warrant further investigation. Considering the critical role of CD4^+^ CART cells in sustaining anti-tumor responses and long-term persistence, adjusting cell dosages may be advisable before clinical translation of single CD4^+^ CART cell treatment.

Mechanistically, the secretome of CD4^+^ CARTs was more potent in stimulating the anti-leukemic activity of CARTs with a more pronounced effect on CD8^+^ CARTs leading to a shift of the CD4/CD8 ratio within the CART products in favor of CD8^+^ CARTs over time. CART products with increasing content of CD4^+^ CARTs secreted higher levels of type 1 and type 2 cytokines, suggesting that both types of immune response play a relevant role for their functionality. In fact, it has been shown that type 2 immune responses are associated with long-lasting remission and that IL-4 and IL-10 may lead to enhanced effector function and improved metabolic fitness of CART cells.^36^^,^^37^^,^^38^^,^^39^

In summary, our data demonstrate the superior anti-leukemic efficacy of pure CD4^+^ CART cells compared with mixed products across multiple CAR specificities and experimental systems. We identify antigen competition as a central mechanism constraining CD4^+^ CART cell function, with CD8^+^ CART cells limiting the magnitude of CD4^+^ activation through competitive consumption of shared antigenic resources. Concurrently, CD4^+^ CART cells in mixed cultures acquire a type I interferon-associated stress signature and pro-apoptotic programming, representing a parallel axis of dysfunction that may compound the consequences of restricted antigen access. Together, these mechanisms—competitive exclusion from optimal activation and interferon-mediated functional erosion–provide a comprehensive framework for understanding CD4^+^ CART cell impairment in mixed products. These findings suggest that the depletion of CD8 CART cells may be key to optimizing the efficacy of CART cell therapeutics. Our results warrant testing of pure CD4^+^ CART products in early-phase clinical trials carefully designed to evaluate both efficacy and the safety profile of this approach.

## Materials and methods

### Generation of CART cells

Retroviral vector production and T cell transduction have been described previously.^40^^,^^41^ In brief, 293T cells were transfected with packaging plasmids (PegPam, RD114) and the SFG vector containing the CD70.CAR, third-generation CD19.CAR, EphA2.CAR, GD2.CAR, CD33.CAR, CLL-1.CAR, second-generation CD19.CAR, or short-spacer CD19.CAR construct, respectively (Figures S1A, S1B, S2A, S2B, S5A, and S5B). All the CAR constructs used is this study were listed in Table S2. The CD70.CAR consisted of human CD27, the only known ligand of CD70 fused to CD3z chain and which has been extensively described in our previous publication.^41^ The long-spacer CD19.CAR, which incorporated the murine FMC63 scFv, has been utilized in a clinical trial (NCT03676504).^42^ For the EphA2 and GD2 targets, the 4H5 and 14G2a scFvs were used for CAR transgenes as described previously.^43^^,^^44^ The CD33-specific scFv was derived from gemtuzumab ozogamicin antibody clone p67.7 and was illustrated in our previous work.^45^ The CLL-1-target CAR utilized the h6E7 scFv, a humanized antibody-derived fragment. The short-spacer CD19.CAR was described in detail in the subsequent section xenograft model. The retroviral supernatant was collected after 48 and 72 h. Peripheral blood mononuclear cells (PBMCs) were isolated from the peripheral blood via density gradient centrifugation. T cells were then stimulated on CD3 (1 μg/mL; Biolegend) and CD28 (1 μg/mL; Biolegend) antibody-coated, non-tissue culture-treated 24-well plates on day 0. From day 1 onward, recombinant human interleukin 7 (IL-7; 10 ng/mL; R&D Systems) and human interleukin 15 (IL-15; 5 ng/mL; R&D Systems) were added to cultures. After 48 h of expansion in complete medium, which consisted of 45% RPMI-1640 (Thermo Fisher Scientific, Waltham, MA), 45% Click’s medium (FujiFilm; Irvine Scientific, Santa Ana, CA), 2 mM GlutaMAX-I CTS (Thermo Fisher Scientific, Waltham, MA), and 10% fetal bovine serum (Thermo Fisher Scientific, Waltham, MA), the T cells were transduced in 24-well plates coated with RetroNectin (Takara) without CD3 and CD28 antibodies. CART cells were transferred from 24-well plates to T75 flasks for further expansion on day 5, with fresh cytokines-supplemented complete medium provided every 3 days. On day 12, FACS was employed to isolate pure CD4^+^ and CD8^+^ CART cells. The two separate subsets were allowed to rest for 2–3 days and formulated to specific CD4/CD8 ratio prior to being used for *in vitro* or *in vivo* experiments.

### Cells and culture conditions

De-identified apheresis products from voluntary healthy donors were sourced from the Heidelberg blood bank. CD19.CART cells, derived from patients in the Heidelberg CART cell trial 1 (HD-CAR-1; NCT03676504), were manufactured in accordance with Good Manufacturing Practice guidelines and stored in a nitrogen tank ready for use. All participants provided signed informed consent and were treated in compliance with the Declaration of Helsinki. 293T cells were acquired from the Deutsche Sammlung von Mikrooganismen und Zellkulturen (DSMZ) and cultured using Iscove’s modified Dulbecco’s medium. Nalm-6, Raji, K562, Molm-13, and HL-60 cell lines were purchased from DSMZ and cultured in RPMI 1640 medium. LAN-1 cells, also obtained from DSMZ, were maintained in RPMI 1640 medium supplemented with 2 mM L-glutamine. A-549 cells, sourced from DSMZ, were cultured in Dulbecco’s modified Eagle medium. All cell lines were cultivated in their respective medium, supplemented with 10% fetal bovine serum and 1% penicillin-streptomycin, within a humidified atmosphere containing 5% CO_2_ at 37°C. All the above-mentioned cell lines were authenticated at DKFZ (German Cancer Research Center) and were free from mycoplasma contamination checked by polymerase chain reaction.

### Flow cytometry

The fluorochrome-conjugated isotype control and antihuman antibodies against CD3, CD4, CD8, CD45RA, CCR7, CD70, CD19, NGFR, PD-1, TIM-3, LAG-3, Ki-67, CD107a, TNF-α, IFN-γ, and annexin V were purchased from BD Bioscience or Biolegend. Biotin-labeled protein L and fluorochrome-labeled streptavidin from Thermo Fisher Scientific and Biolegend were used to identify CAR expression of CD33.CAR, CLL-1.CAR, and long-spacer CD19.CAR (SFG.CD19.CH2-CH3.CD28.4-1BB.CD3zeta). Additionally, to precisely detect the short-spacer CD19.CAR (SFG.CD19.CH3.CD28.4-1BB.CD3zeta) expression, the CD19 CAR Detection Reagent (biotinylated) together with anti-biotin-PE from Miltenyi Biotec was applied. The expression of CD70.CAR and GD2.CAR was assessed by identifying truncated CD19 via an APC-conjugated or PE-conjugated antibody specific to CD19. Similarly, EphA2.CAR expression was ascertained by detecting truncated NGFR using an antibody specific to NGFR, conjugated with either APC or PE. Dead cells were removed from the analysis using either the LIVE/DEAD fixable near-infrared dead cell stain kit (Thermo Fisher Scientific) or 7AAD (BD Biosciences). Fluorescence compensation was conducted for every specific experiment with different FACS panels before cell acquisition in a flow cytometer. Fluorescence minus one control, isotype control, or NT control was included. Flow cytometry data were acquired using BD FACSymphony A3 and BD LSRII cell analyzers and visualized with FlowJo V.10.10.0 software.

### Surface marker staining

Cells were harvested in the FACS tube and washed once with FACS buffer. The supernatant was removed from the cells after centrifuging. If necessary, 5 μl of Fc Receptor Blocking Solution (Biolegend) per million cells was added in 100 μL staining volume prior to staining with antibody of interest and incubated at room temperature for 5–10 min. A cocktail of fluorochrome-labeled antibodies against the surface markers of interest was prepared and added to cells and mixed well. After incubation for 30 min at 4°C in the dark, cells were washed with FACS buffer. Finally, cells were resuspended in 400 μL of FACS buffer supplemented with 7-AAD Viability Staining Solution for measurement.

### Intracellular cytokine staining

CD70.CART, CD19.CART, and CD33.CART cells were seeded into a 96-well U-bottom plate. The cells were then stimulated with either the respective target tumor cells or non-target K562 cells in the presence of monensin, brefeldin A, and CD107a antibody for 5 h at 37°C. Afterward, the cells were washed with PBS and stained with NEAR-IR for 30 min at 4°C in the dark. Finally, surface markers were stained for 20 min at 4°C in the dark. The cells were fixed and permeabilized at room temperature following the instructions of the Miltenyi Fixation/Permeabilization Solution Kit. Antibodies were used to stain cytokines IFN-γ and TNF-α for 30 min in the dark at room temperature. Lastly, the cells were washed and resuspended in FACS buffer for acquisition.

### CFSE staining

CART cells were resuspended in a CFSE (carboxyfluorescein succinimidyl ester) staining solution (Biolegend) comprising 1,000 μL of PBS and 1 μL of a 5 mM CFSE stock. This cell preparation was then incubated for 20 min at 37°C in the dark. The staining reaction was halted by the introduction of complete medium, followed by two additional washes with the medium. For the assay, 1 × 10^6^ CFSE-stained cells were allocated per well into a 24-well plate and were then stimulated by X-ray-irradiated target tumor cells at a 1:1 ratio. The proliferative response of the CART cells was evaluated after a 5-day stimulation period.

### IncuCyte live-cell cytotoxicity assay

To assess the functional persistence of culture condition-induced phenotypes, CD4^+^ CART cells were re-sorted from either pure CD4^+^ cultures (PCD4) or from 1:1 mixed CD4^+^/CD8^+^ isospecific cultures (MCD4) on day 12 of serial co-culture using a BD FACSAria III cell sorter. Re-sorted CD4^+^ CART cells were then co-cultured with fresh Nalm-6 target cells at an E:T ratio of 1:1 in the absence of CD8^+^ CART cells. Cells were seeded at a density of 2 × 10^4^ cells per well for both effector and target populations in 96-well plates. Real-time cytotoxicity was measured using the IncuCyte S3 Live-Cell Analysis System (Sartorius). Nalm-6 target cells stably expressed zsGreen for identification; dead cells were detected by propidium iodide (1 μg/mL) uptake. Images were acquired every 2 h for 96 h, and dead tumor cells (zsGreen^+^/propidium iodide^+^) were quantified using IncuCyte analysis software. Cytotoxicity was expressed as fold change in dead tumor cells relative to t = 0. Cumulative killing was calculated as the AUC from 0 to 96 h. Statistical comparison between PCD4 and MCD4 conditions was performed using a paired two-tailed *t* test (*n* = 6 independent healthy donors).

### Co-culture assay

An illustrative scheme of the experimental approach is presented in Figure S1C. CART cells were co-cultured with tumor cells at the appropriate E:T ratio in 96-well plates without the addition of external cytokines. At the end of each round of the co-culture, one well for each condition was harvested, and the total count of T cells and tumor cells was determined by flow cytometry using CountBright counting beads. CART cells were subjected to a repetitive challenge with the same number of fresh tumor cells that were initially added in predefined intervals until the CARTs lost their ability to eliminate tumor cells or failed to further proliferate and subsequently disappeared. Conditions with strong CART proliferation were subsequently transferred to 48-, 24-, or 12-well plates to prevent overcrowding.

### Cytokine release assay

Supernatants were collected 24 h after initiation of the co-culture. To quantify cytokines in the supernatant, bead-based multiplex LEGENDplex analysis (BioLegend) was performed following the manufacturer’s instructions. Standard curves generated using the protein standards provided in the kit. A range of proinflammatory cytokines, including IL-2, IL-4, IL-6, IL-10, IL-17A, IFN-γ, TNF-α, soluble Fas, soluble FasL, granzyme A, granzyme B, perforin, and granulysin, were measured. The analysis was conducted on the BD FACSCantoII flow cytometer. Data were interpreted by the LEGENDPlex V8.0 software (Biolegend).

### Xenograft model

*In vivo* experiments were conducted in accordance with a protocol endorsed by the federal and Institutional Animal Care and Use Committee (IACUC). The female and male NOD.Cg-Prkdc^scid^ lL2rg^tm1Wjl^/SzJ (NSG) mice, aged 6 to 10 weeks, were acquired from the Heidelberg University Interfaculty Biomedical Research Facility (IBF) breeding colony and maintained in IBF during the experiments. Animals were administered injections of tumor cells and T cells intravenously through the tail vein, as detailed in the results and supplemental information sections. Subsequent measurements of tumor mass or T cell expansion were taken regularly by bioluminescent imaging, using the *in vivo* imaging system IVIS Lumina II (Caliper LifeScience, Hopkinton, MA). The mice were euthanized when they reached predefined endpoint criteria complied with IACUC.

Rather than employing the CD19.CAR construct with a hinge-CH2-CH3 spacer (long spacer), which was utilized in both the *in vitro* experiments and the Heidelberg CART cell trial 1 (HD-CAR-1; NCT03676504),^46^ a short-spacer CD19.CAR construct featuring a hinge-CH3 was used to modify the T cells for the murine leukemia xenograft treatment (Table S2). Previous observations, such as those reported by Almasbak et al. in 2015, have identified that the CH2 domain in commonly used IgG1-Fc spacers can bind to soluble mouse Fcγ-receptor I, leading to off-target T cell activation directed at murine macrophages and consequently diminishing anti-leukemia activity.^47^ To address this issue, our study adopted a short-spacer CAR construct that omits the CH2 region in the NSG mouse model.

### Bulk RNA sequencing of sorted CART co-cultures

CD70-directed and CD33-directed CART cells were generated from healthy donor PBMCs as described in the main methods. For transcriptomic analysis, CART cells were co-cultured with their respective target cells (Molm-13 for CD70.CART; HL-60 for CD33.CART) at an E:T ratio of 1:1 with fresh target cells added every 3 days. On day 12 of co-culture, CD4^+^ CART cells were isolated by FACS from two conditions: (1) pure CD4^+^ CART cultures (PCD4) and (2) 1:1 mixed CD4^+^/CD8^+^ CART cultures (MCD4). For each CAR construct (CD70 and CD33), three independent healthy donors were used (*n* = 3 per construct; *n* = 6 total donors). Sorting was performed based on CD4 and CD8 surface expression using a BD FACSAria III cell sorter.

Total RNA was extracted from sorted cells using the RNeasy Mini Kit (Qiagen) according to manufacturer’s instructions. RNA quality was assessed by the sequencing provider. Only samples achieving an RNA integrity number >8 were accepted. Library preparation and sequencing were performed by BGI Genomics Co. (Shenzhen, Guangdong, China) using their proprietary DNBSEQ sequencing platform. Paired-end sequencing (150 bp) was performed with an average depth of 30 million reads per sample.

Raw sequencing reads were processed by BGI Genomics using their standard pipeline, which included quality filtering, adapter trimming, and alignment to the human reference genome (GRCh38). Gene-level read counts were quantified and provided for downstream analysis. Differential expression analysis comparing PCD4 versus MCD4 was performed separately for each CAR construct using DESeq2 (version 1.38.0) in R (version 4.2.0). Genes with fewer than 10 counts across all samples were filtered prior to analysis. *p* values were adjusted for multiple testing using the Benjamini-Hochberg method. Differentially expressed genes were defined using thresholds of adjusted *p* < 0.05 and |log2FC| ≥ 1. GSEA was performed using the clusterProfiler package (version 4.6.0) with genes ranked by log2 fold change (PCD4/MCD4). Gene symbols were mapped to Entrez IDs using the org.Hs.eg.db annotation package. Hallmark gene sets were obtained from the Molecular Signatures Database (MSigDB) via the msigdbr package (version 7.5.1). GSEA was performed separately for CD70 CART and CD33.CART datasets using a permutation-based approach with 1,000 permutations. Pathways were considered significantly enriched at FDR <0.05. Consensus pathways were identified as those reaching significance (FDR < 0.05) in both CAR construct analyses. The processed gene count matrices generated in this study have been deposited in the Zenodo repository and are publicly available under accession https://doi.org/10.5281/zenodo.18421095.

### Label-free proteome measurement

FACS-sorted CD4^+^ CART cells from the co-culture condition were thoroughly washed in plain PBS, lysed in 1%SDC buffer (1% SDC, 100 mM Tris [pH 8.5], 40 mM CAA, and 10 mM TCEP), incubated on ice for 20 min, boiled at 95°C, and sonicated for 5 min on a Biorupter plus as described previously.^48^ Samples were digested with trypsin and LysC for 16 h at 37°C. Digestion was stopped by adding 5× volumes of isopropanol/1% TFA and vortexing vigorously. The peptides were de-salted on equilibrated styrene divinylbenzene-reversed phase sulfonated (SDB-RPS) StageTips, washed with isopropanol/1% TFA and 0.2% TFA, and eluted with 60 μL of elution buffer (1.25% ammonia, 80% ACN). The dried elutes were resuspended in MS loading buffer (3% ACN, 0.3% TFA) and stored at −20°C until MS measurement.

400 ng peptides were loaded onto a nanoElute system (Bruker Daltonics, Bremen, Germany) coupled with a TIMS TOF HT mass spectrometer (Bruker Daltonics, Bremen, Germany) using a CaptiveSpray nano-electrospray ion source. Peptides were separated on an IonOpticks Aurora 25-cm column using a binary buffer system: buffer A (0.1% formic acid, 2% ACN) and buffer B (0.1% FA in 99.9% ACN), using a 120-min gradient starting from 2% buffer B, increasing to 12% in 60 min, 20% in 30 min, 30% in 10 min, and finally reaching 85% in 10 min, which was held for an additional 10 min at a flow rate of 0.3 μL/min. DDA-PASEF mode was employed, and MS1 spectra were recorded from 150 m/z to 1,700 m/z, with TIMS functioning at scan range of 100–1,700 m/z, ramp time of 100 ms, duty cycle of 100%, cycle time of 100.00 ms, and spectra rate of 9.43 Hz.

### MS data analysis and bioinformatics

MS raw files were processed using MaxQuant (version 1.5.5.2) supported by Andromeda search engine against the UniProt human reference fasta (version 2016). MaxQuant default settings were employed for identification and quantification, allowing a maximum of 2 missed cleavages. The minimal peptide length was set to 7. Label-free quantification (LFQ) was performed using the MaxLFQ algorithm implemented in MaxQuant, with the following settings: a minimal ratio count of 2 and fast LFQ enabled. The peptide and protein FDR was set to 1% to ensure high confidence in the identified peptides and proteins.

The protein group file generated from MaxQuant software was further analyzed in Perseus (version 2.0.7.0). Values were log2 transformed, and data were pre-cleaned by filtering out contaminants, reverse, and only identified by site modifications before statistical analysis. Enrichment analysis and keywords were performed by comparing the protein expressions between groups. Paired sample *t* tests and multi-sample *t* tests were used to compare between two groups with a permutation-based FDR cutoff of 5%. The proteome data has been submitted to the PRIDE database (PRIDE: PXD060508).

### Statistical analysis

Statistical analysis was performed using Prism 10 software (GraphPad Software, San Diego, CA). For analyzing continuous variables across three or more groups, one-way analysis of variance was employed. When comparing two groups, the analysis was carried out using either a *t* test or a Wilcoxon rank-sum test. Survival times resulting from the injection of tumor cells in mouse experiments were analyzed through the use of Kaplan-Meier curves and the Mantel-Cox log rank test. *p* values <0.05 were defined as statistically significant.

### Data and code availability

Data generated from proteome analysis are deposited in the PRIDE database (PRIDE: PXD060508). The code used for the analyses is available upon reasonable request.

## Acknowledgments

We thank Dr. Volker Eckstein and Stefanie Hofmann for assistance with the fluorescence-activated cell sorting. We thank all patients for providing their specimens. The project was funded by the 10.13039/501100001659Deutsche Forschungsgemeinschaft (DFG, German Research Foundation) – SFB 1709/1 2025–533056198.

## Author contributions

Q.C., D.S., T.S., M.S., and A.K.J. designed the research; Q.C., D.S., and T.S. analyzed and interpreted the data and wrote the paper; Q.C., D.S., H.Y., L.H.M., D.D., D.S., M.S., B.L.H., and J.M.U. performed the experiments; L.W., A.S., C.M.-T., and A.K.J. contributed to the interpretation of the results.

## Declaration of interests

The authors declare no competing interests.

## Supplemental information

Document S1. Tables S1 and S2, Figures S1–S15

Document S2. Article plus supplemental information
