# Supplementary material for: Depletion of CD8+ CART cells leads to superior anti-tumor efficacy of pure CD4+ CART cells against acute leukemias
Source: Mol Ther. 2026 Jun 3;34(8):4687–707. doi: 10.1016/j.ymthe.2026.05.027 (PMC13464297; doi:10.1016/j.ymthe.2026.05.027)
Supplement: Supplementary file 1 [file mmc1.pdf]

YMTHE, Volume *34*

Supplemental Information

Depletion of CD8$^+$ CART cells leads to superior anti-tumor efficacy of pure CD4$^+$ CART cells against acute leukemias

Qian Chen, David Sedloev, Hao Yao, Lianghao Mao, Dominic Depke, Lei Wang, Marina Scheller, Bailin He, Yi Liu, Julia M. Unglaub, Anita Schmitt, Ashok Kumar Jayavelu, Carsten Müller-Tidow, Michael Schmitt, and Tim Sauer

Table S1. The baseline information and treatment characteristics of patients from HD- CAR1 clinical trial.

Patient	Age	Diagnosis	$CD4^{+}CART:CD8^{+}CART$	Response
S1-010	36	ALL	68% : 32%	PD
S1-016	32	ALL	14% : 86%	CR*
S2-007	61	CLL	70% : 30%	SD
S2-009	59	tFL/DLBCL	74% : 26%	SD
S2-010	48	FL	14% : 86%	PR
S2-011	63	MCL	43% : 57%	CR

ALL: acute lymphoid leukemia; CLL: chronic lymphocytic leukemia; MCL: mantle cell lymphoma; FL: follicular lymphoma; tFL/DLBCL: transformed follicular lymphoma into diffuse large B-cell lymphoma. CR: complete response, minimal residual disease+ (MRD+); CR*: complete response, minimal residual disease- (MRD-); PD: progressive disease; SD: stable disease; PR: partial response.

Table S2. Profile of CAR constructs used in this study.

CAR Construct	Target	Co-stim domain	Reference
SFG.CD27.CD3zeta.IRES.tCD19	CD70	CD27	[41]
SFG.FMC63.CH2CH3.CD28.4-1BB.CD3zeta	CD19	CD28, 4-1BB	[42]
SFG.4H5.CH2CH3.CD28.CD3zeta.T2A.tNGFR	EphA2	CD28	[43]
SFG.14g2a.CH2CH3.CD8a.4-1BB.CD3zeta.IRES.tCD19	GD2	4-BB	[44]
SFG.CD33scFv.CH2CH3.CD28.4-1BB.CD3zeta	CD33	CD28, 4-1BB	[45]
SFG.h6E7.CH3.CD28.CD3zeta	CLL-1	CD28	
SFG.FMC63.CH2CH3.CD28.CD3zeta	CD19	CD28	
SFG.FMC63.CH2CH3. 4-1BB.CD3zeta	CD19	4-1BB	
SFG.FMC63.CH3.CD28.4-1BB.CD3zeta	CD19	CD28, 4-1BB	

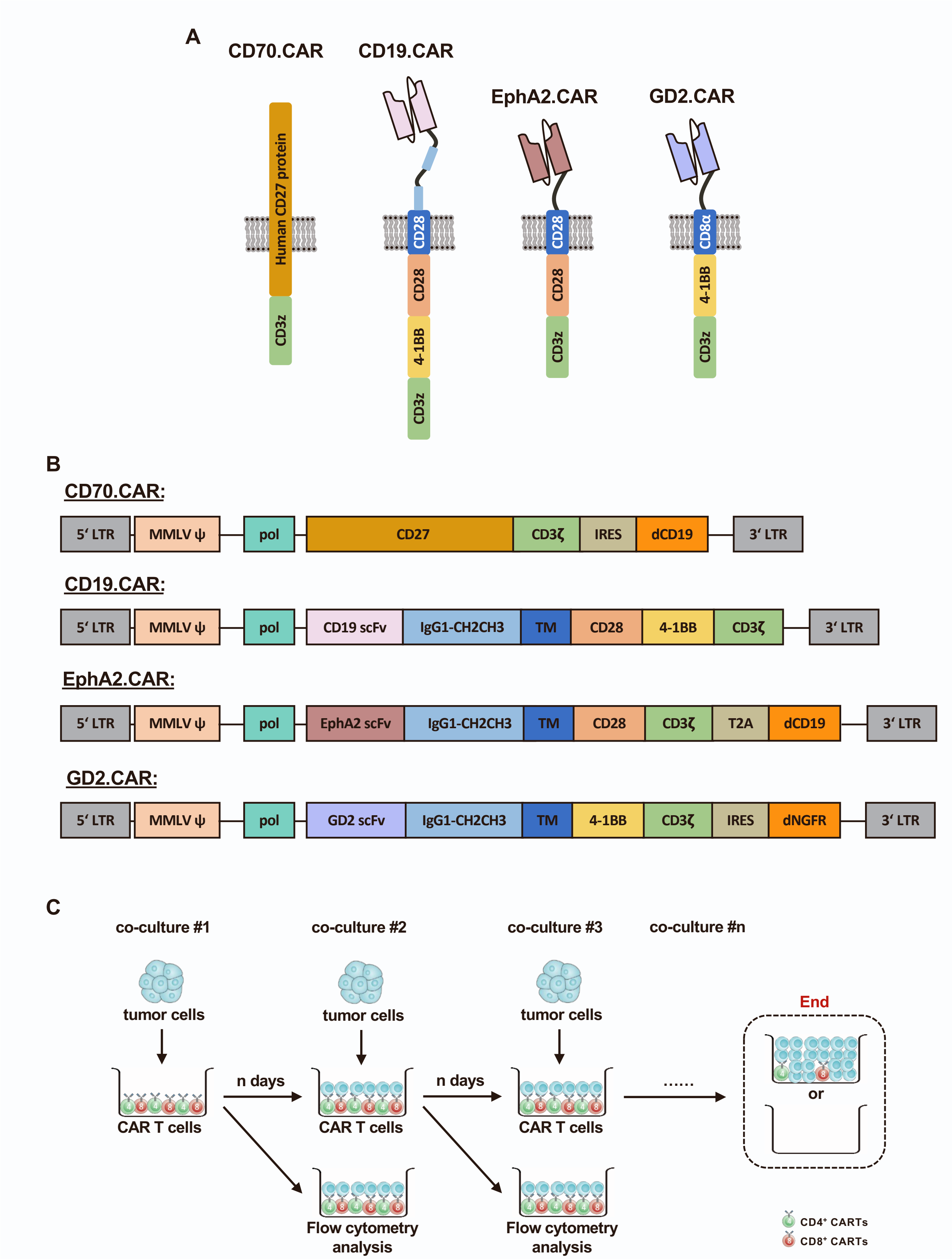

Figure S1. Structural composition of CAR constructs and experimental set-up of the co-

culture assay.

(A) Schematic representation of the structural composition of the CD70.CAR, CD19.CAR, EphA2.CAR and GD2.CAR. (B) Schematic outline of the retroviral vectors used for the generation of CD70.CART, CD19.CART, EphA2.CART and GD2.CART cells. (C) Schematic representation of the experimental design of the serial co-culture assay. CART cells were co-cultured with tumor cells at appropriate Effector-to-Target (E:T) ratios in plates without the addition of external cytokines. A single well was harvested on the pre-specified day of the co-culture and the absolute tumor and T cell numbers at the end of each co-culture were determined by flow cytometry using CountBright counting beads. The same number of fresh tumor cells as in co-culture #1 were repeatedly added to the co-culture until CARTs failed to kill the tumor cells or failed to persist after tumor cell killing.

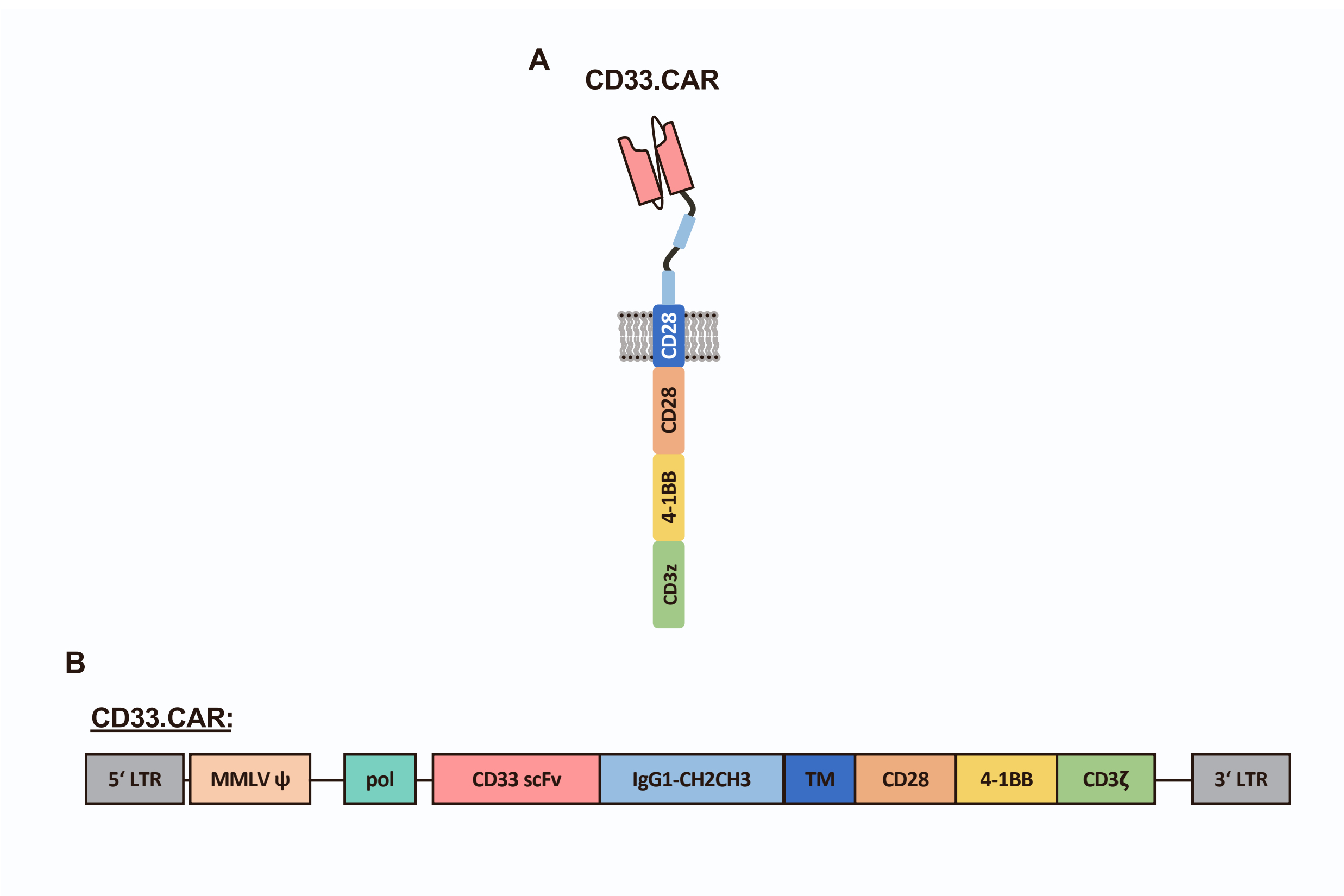

Figure S2. Structural composition of CD33.CART construct.

(A) Schematic outline of the structural composition of CD33.CAR. (B) Schematic outline of the retroviral vectors used for the generation of CD33.CART cells.

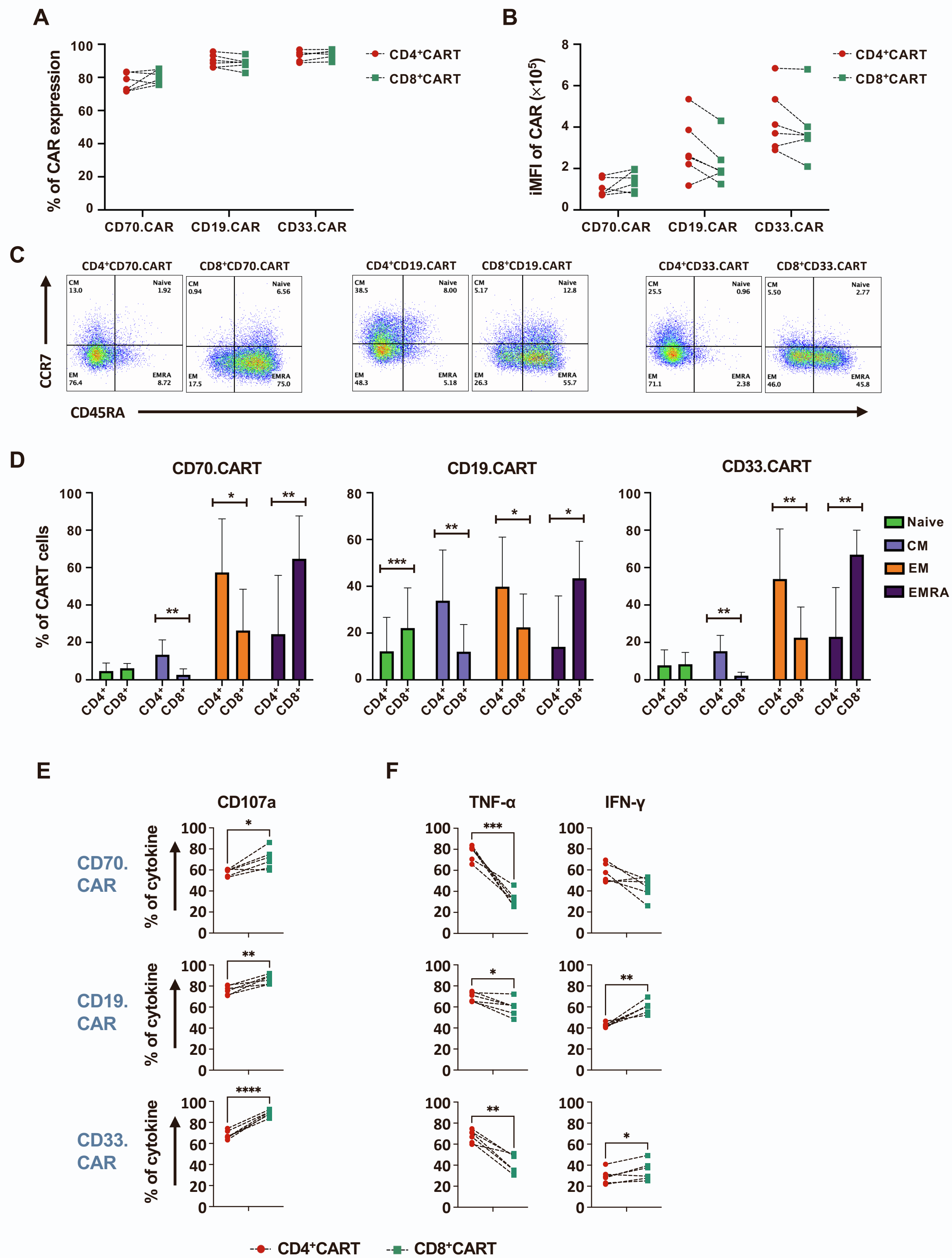

Figure S3. Phenotypical characterization of CD4^{+} and CD8^{+} CART cells.

(A-B) Expression of the CD70 CAR, the CD19 CAR and the CD33 CAR. (A) Percentage of CAR expressing $CD4^+$ and $CD8^+$ CARTs and (B) the integrated mean fluorescence intensity of CAR expression (iMFI) as determined by flow cytometry on day 12 after T-cell activation: $CD3^+CD4^+CAR^+$ vs $CD3^+CD8^+CAR^+$ ($n = 6$, P=ns (not significant), paired t test). The expression of CD70.CAR was determined by detection of a truncated CD19 using an APC-conjugated CD19 specific antibody. The CD19.CAR and the CD33.CAR were detected by staining with biotin-labeled protein L followed by subsequent staining with APC-conjugated streptavidin. (C) Representative dot plots depicting the immunophenotype of CD70.CART, CD19.CART and CD33.CART on day 16 after T-cell activation of T-cell transduction by flow cytometry. (D) Immunophenotypic analysis of $CD4^+$ and $CD8^+$ CART cells on day 16 after T-cell activation (CD4 vs CD8, naive: CD45RA+CCR7+; central memory [CM]: CD45RA–CCR7+; effector memory [EM]: CD45RA−CCR7−; EM T cells re-express CD45RA [EMRA]: CD45RA+CCR7−) ($n = 6$, paired t test, data represent mean ± SD, *$P < 0.05$; **$P < 0.01$; ***$P < 0.001$). (E) CD107a expression and (F) TNF-α and IFN-γ secretion 4 hours after stimulation with antigen positive tumor cells (E:T ratio 1:1) as determined by intracellular staining followed by flow cytometry. ($n = 6$, paired t test, *$P < 0.05$; **$P < 0.01$; ***$P < 0.001$; ****$P < 0.0001$).

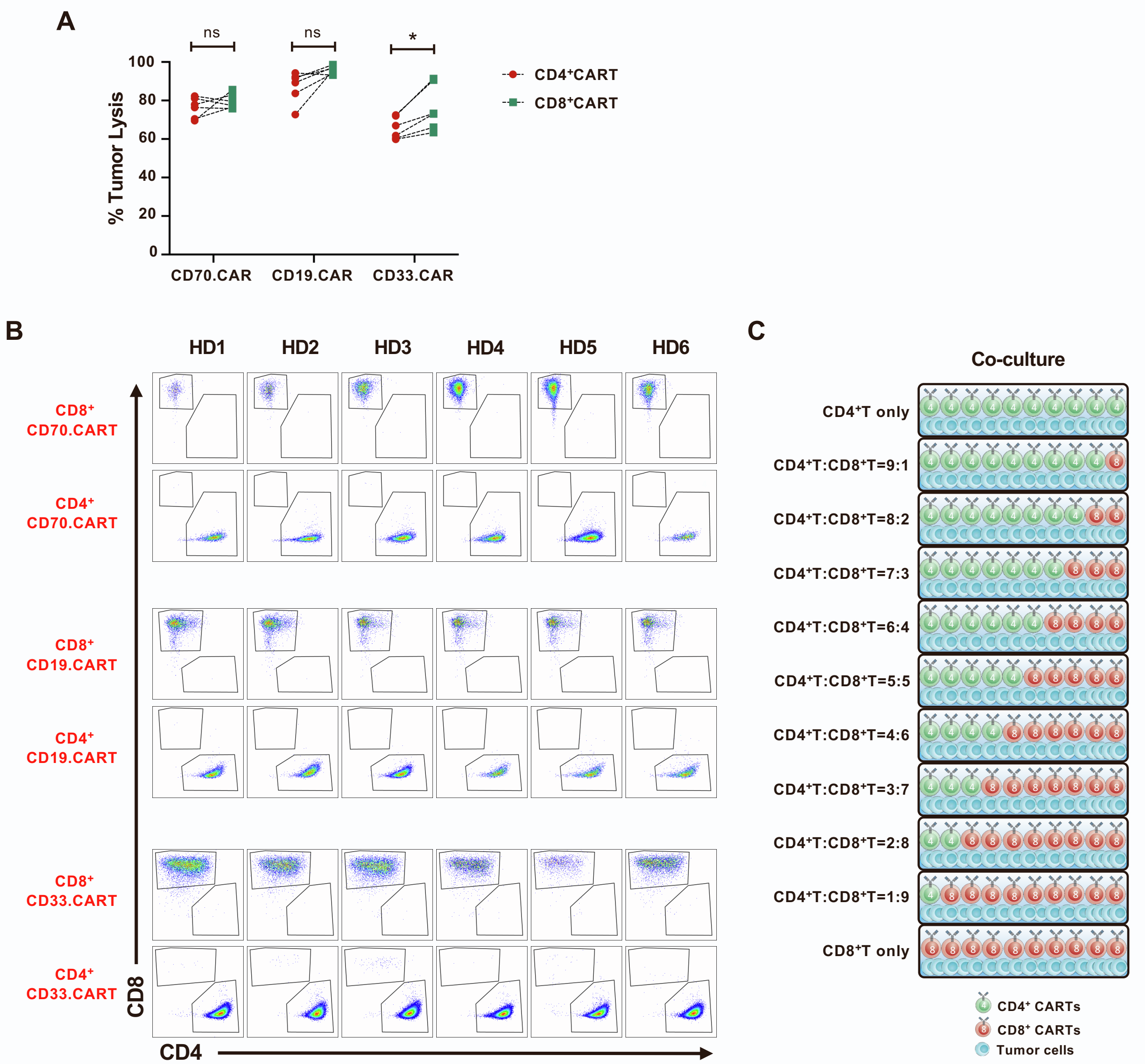

Figure S4. Short-term cytotoxicity of CD4$^+$ and CD8$^+$ CART cells and generation of CART products with distinct CD4$^+$/CD8$^+$ T-cell ratios to be used in serial co-culture assays.
(A) Short-term cytotoxicity of CARTs was determined after co-culturing CD70.CARTs, CD19.CARTs and CD33.CARTs for 24 hours with Molm-13, Nalm-6, HL-60 cells, respectively, at an E:T ratio of 1:2. The absolute number of residual tumor numbers were determined by flow cytometry using CountBright counting beads (n = 6, paired t test, *P < 0.05; ns, not significant). (B) Representative dot plots of CD4$^+$ and CD8$^+$ CD70.CARTs, CD19.CARTs and CD33.CARTs from 6 healthy donors (HD) after fluorescence-activated cell sorting (FACS) on day 12 of T-cell transduction. (C) These CARTs were then used to set-up serial co-culture assays with target antigen expressing tumor cells at the displayed CD4$^+$/CD8$^+$ CART ratios.

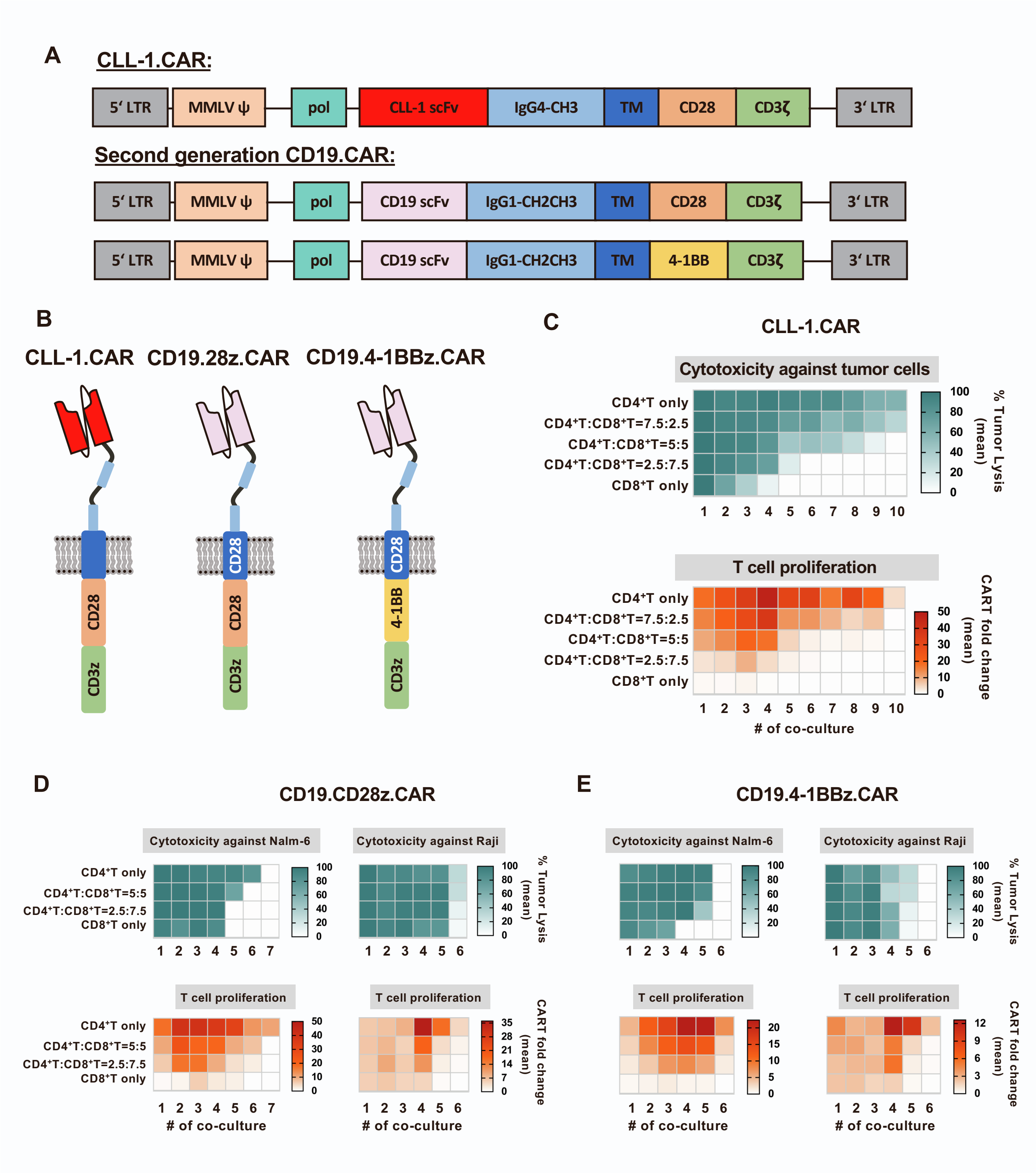

Figure S5. CD4$^+$ CART cell functional superiority is independent of co-stimulatory domain. (A-B) Schematic outline of the structural composition of a second-generation CLL-1.CAR containing the CD28 co-stimulatory domain and second-generation CD19-directed CARs containing either the CD28 or 4-1BB co-stimulatory domain. (C) CLL-1.CARTs and HL-60 cells expressing CLL-1 were cocultured at an E:T ratio of 1:3 and fresh tumor cells were added every 3 days. (D-E) Serial co-culture assays comparing CD19 CART products at various CD4$^+$/CD8$^+$ ratios against Nalm-6 (D) and Raji (E) target cells. CART cells were co-cultured at an E:T ratios of 1:1 (Nalm-6) or 2:1 (Raji), with fresh tumor cells added every 3 days. The absolute tumor and

T cell numbers at the end of each co-culture were determined by flow cytometry using CountBright counting beads (*n* = 6). Double-gradient heat map of tumor cell killing (blue, 100% target cell lysis; white, 0% target cell lysis); Double-gradient heat map of T cell proliferation (red, highest proliferation value; orange, baseline proliferation value; white, no proliferation).

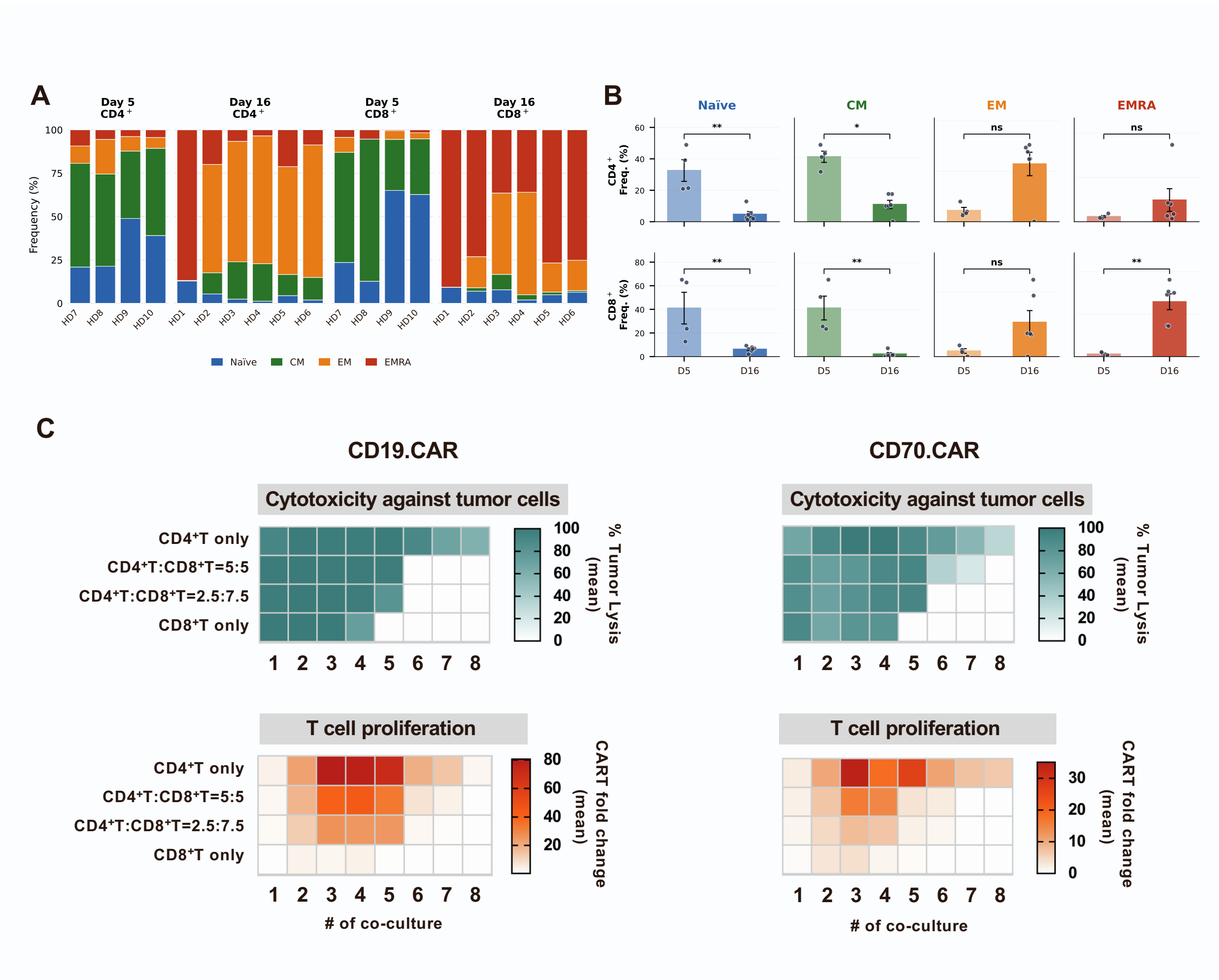

Figure S6. CD4$^+$ CART cell functional superiority is maintained with a shortened manufacturing protocol.

(A) T cell differentiation phenotype distribution of CD4$^+$ and CD8$^+$ CART cells at Day 5 (shortened protocol) and Day 16 (standard protocol) of manufacturing. Stacked bar graphs show the frequency of naïve (CD45RA+CCR7+), central memory (CM; CD45RA−CCR7+), effector memory (EM; CD45RA−CCR7−), and terminally differentiated effector memory (EMRA; CD45RA+CCR7−) subsets for individual healthy donors (HD7–HD10 for Day 5; HD1–HD6 for Day 16). (B) Quantitative comparison of differentiation subset frequencies between Day 5 and Day 16 for CD4$^+$ (upper panels) and CD8$^+$ (lower panels) CART cells (paired t-tests, data represent mean ± SD, *$P < 0.05$; **$P < 0.01$; ns, not significant). (C) Serial co-culture assays with CART products generated using the shortened manufacturing protocol (sorted on Day 5). CD19.CART cells were co-cultured with Nalm-6 cells and CD70.CART cells were co-cultured with Molm-13 cells, both at an E:T ratio of 1:1. Fresh tumor cells were added every 3 days (CD19.CART) or every 4 days (CD70.CART). The absolute tumor and T cell numbers at the end of each co-culture were determined by flow cytometry using CountBright counting beads (n = 4). Double-gradient heat map of tumor cell killing (blue, 100% target cell lysis; white, 0% target cell lysis); Double-gradient heat map of T cell proliferation (red, highest proliferation value; orange, baseline proliferation value; white, no proliferation).

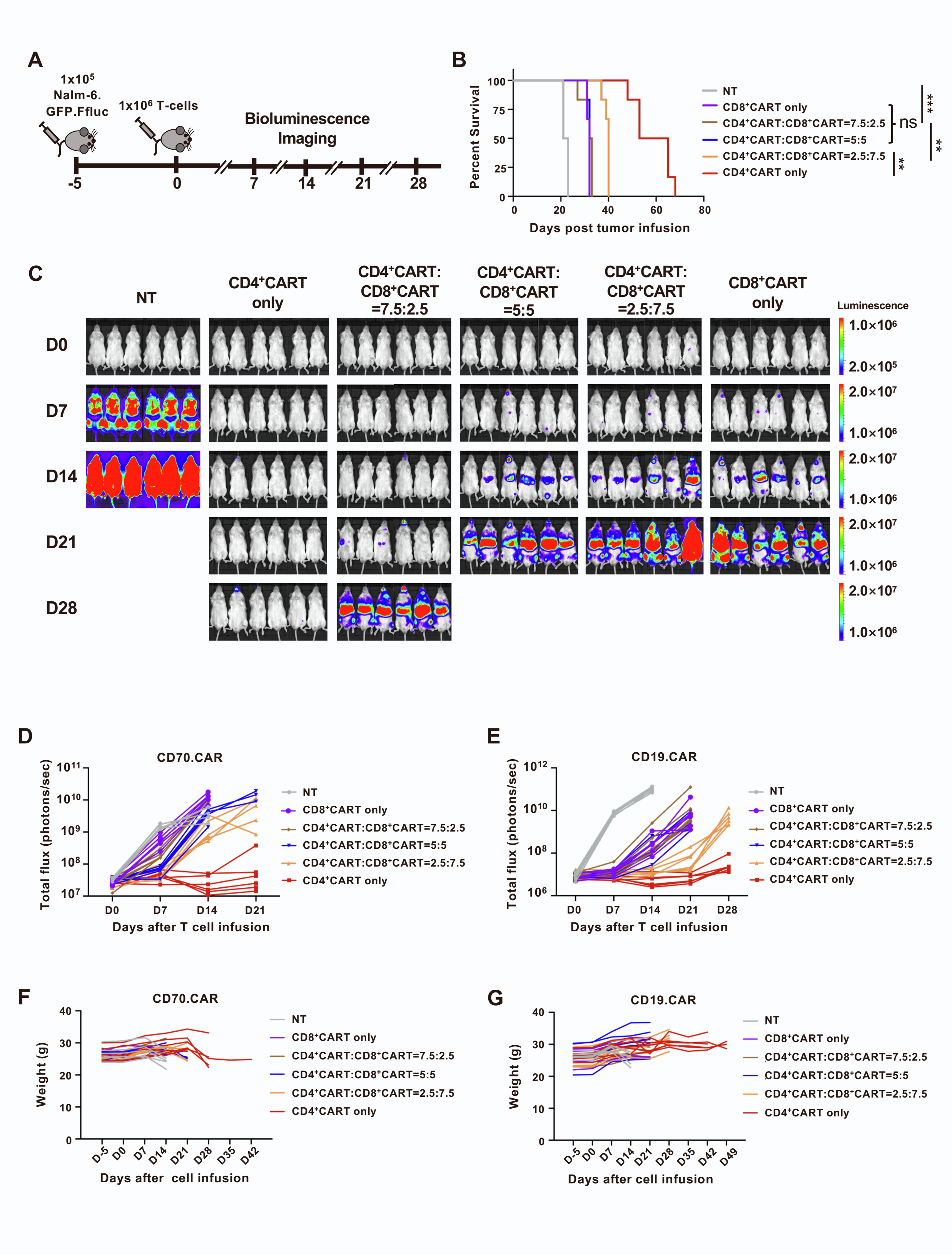

Figure S7. Superior antileukemic activity of CD4⁺ CART cells in murine xenograft models.

(A) Experimental setup of the Nalm-6 xenograft model. Short-spacer CD19.CART cells (1×10^6), depicted in Supplementary Figure 6, and non-transduced (NT) T cells (1×10^6) were administered 5 days after injection of GFP firefly luciferase (Ffluc)-labeled Nalm-6 cells (1×10^5). Bioluminescence imaging (BLI) was performed prior to T cell injection on day 0 to confirm tumor cell engraftment and continued weekly thereafter (*n* = 6 for each treatment group). (B) Kaplan-Meier survival graph of mice treated with either short spacer CD19.CART or NT T cells. The statistical analysis of survival between the treatment groups was performed using the log-rank (Mantel-Cox) test (*n* = 6, **P < 0.01; ***P < .001; ns, not significant). (C) Images depicting the BLI signal derived from Nalm-6 cells on the specified days in mice treated with either NT T cells or short-spacer CD19.CARTs at various $CD4^+/CD8^+$ CART ratios. (D-E) Evolution of total flux measured by BLI for each individual mouse in every treatment group for the CD70.CART model (D) and CD19.CART model (E). The CD70.CART model was shown in Figure 3A-C. (F-G) The body weight of treated mice in the CD70.CART (F) and CD19.CART (G) models was measured as an indicator of their overall condition.

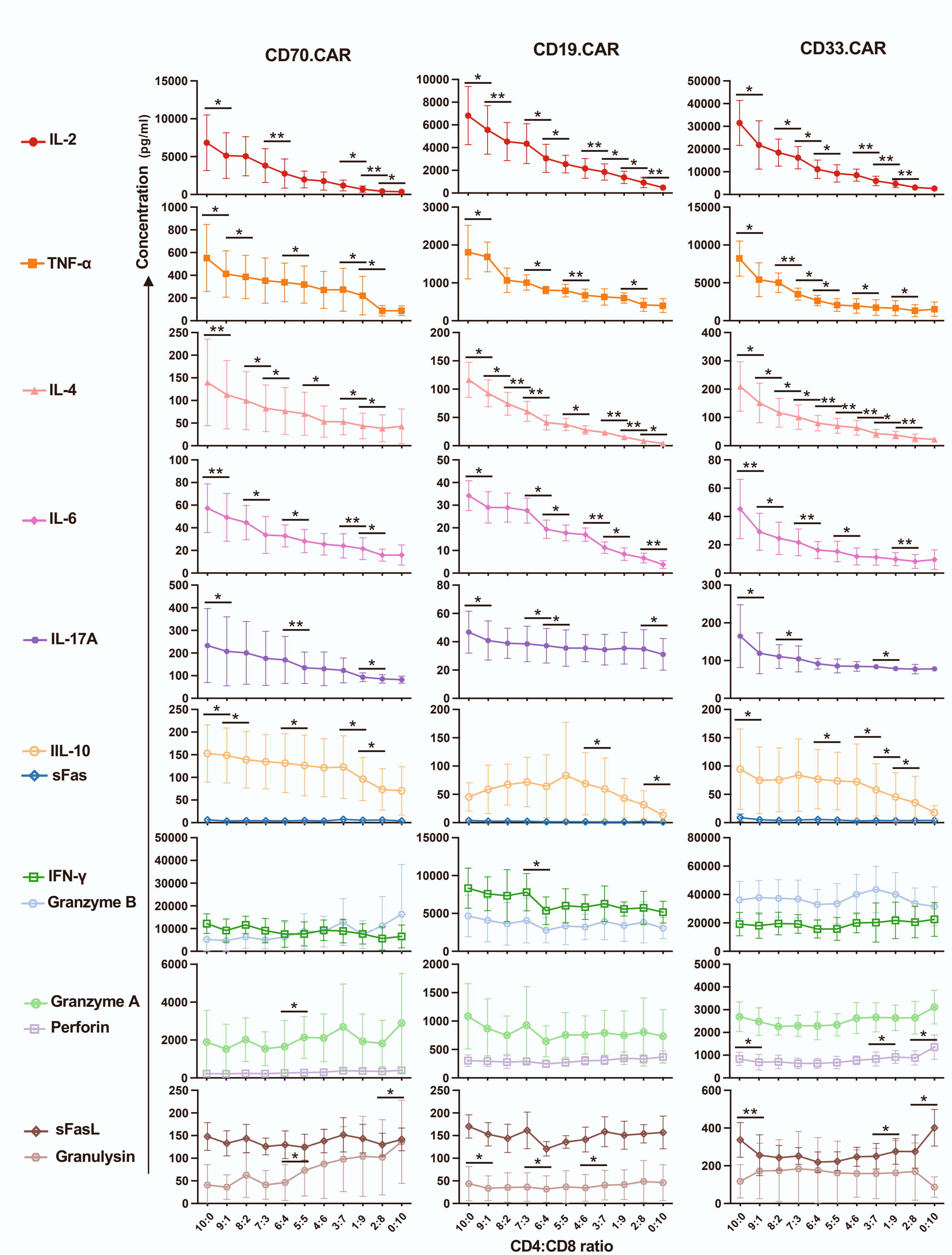

Figure S8. Cytokine release profile of CD19.CART, CD70.CART and CD33.CART products.

Statistical analysis of the cytokine secretion by CD19.CART, CD70.CART and CD33.CART products. (*n* = 6, paired t test, data represent mean ± SD, *P < 0.05; **P < 0.01; ns, not significant).

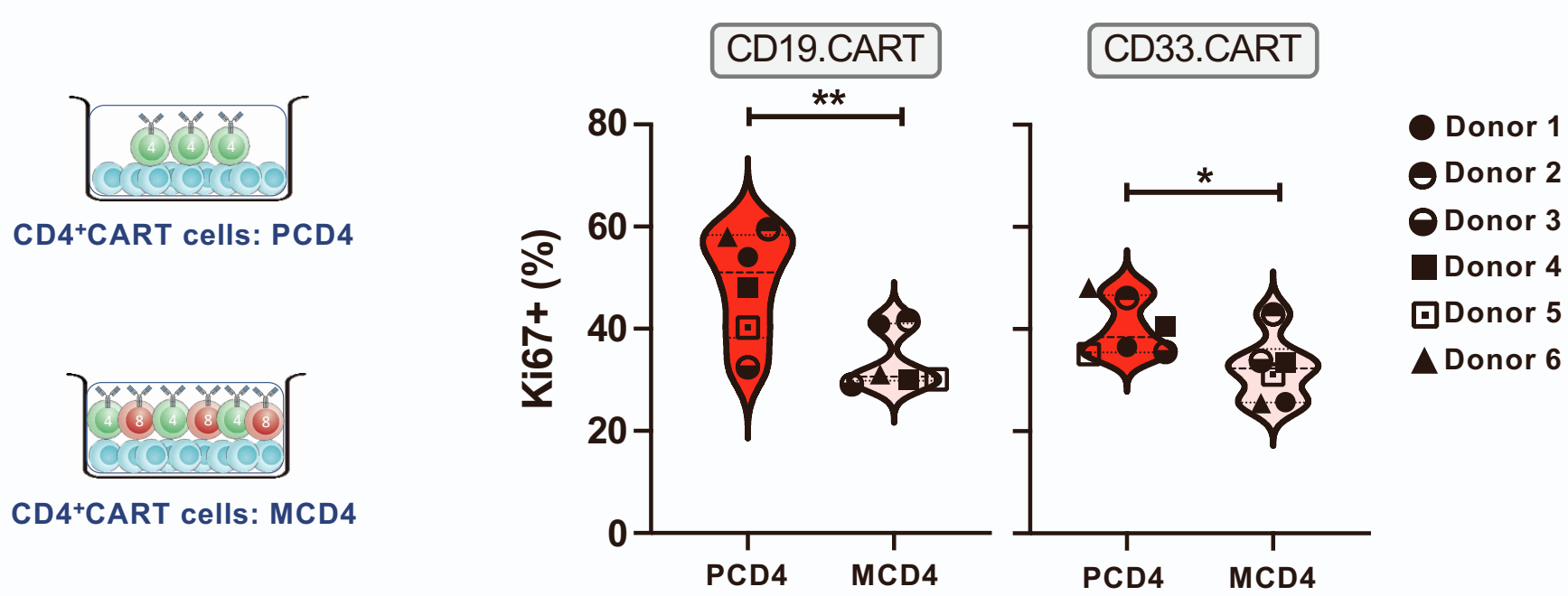

Figure S9. Assessment of Ki67 expression in PCD4 and MCD4 cells.
CD19.CARTs and CD33.CARTs derived from healthy donors were co-cultured with Nalm-6 and HL-60 cells respectively every 3 days at an E:T ratio of 1:3. Ki67 expression on PCD4 and MCD4 CARTs was determined by flow cytometry on day 9 of co-culture (*n* = 6, paired t test, *P < 0.05; **P < 0.01).

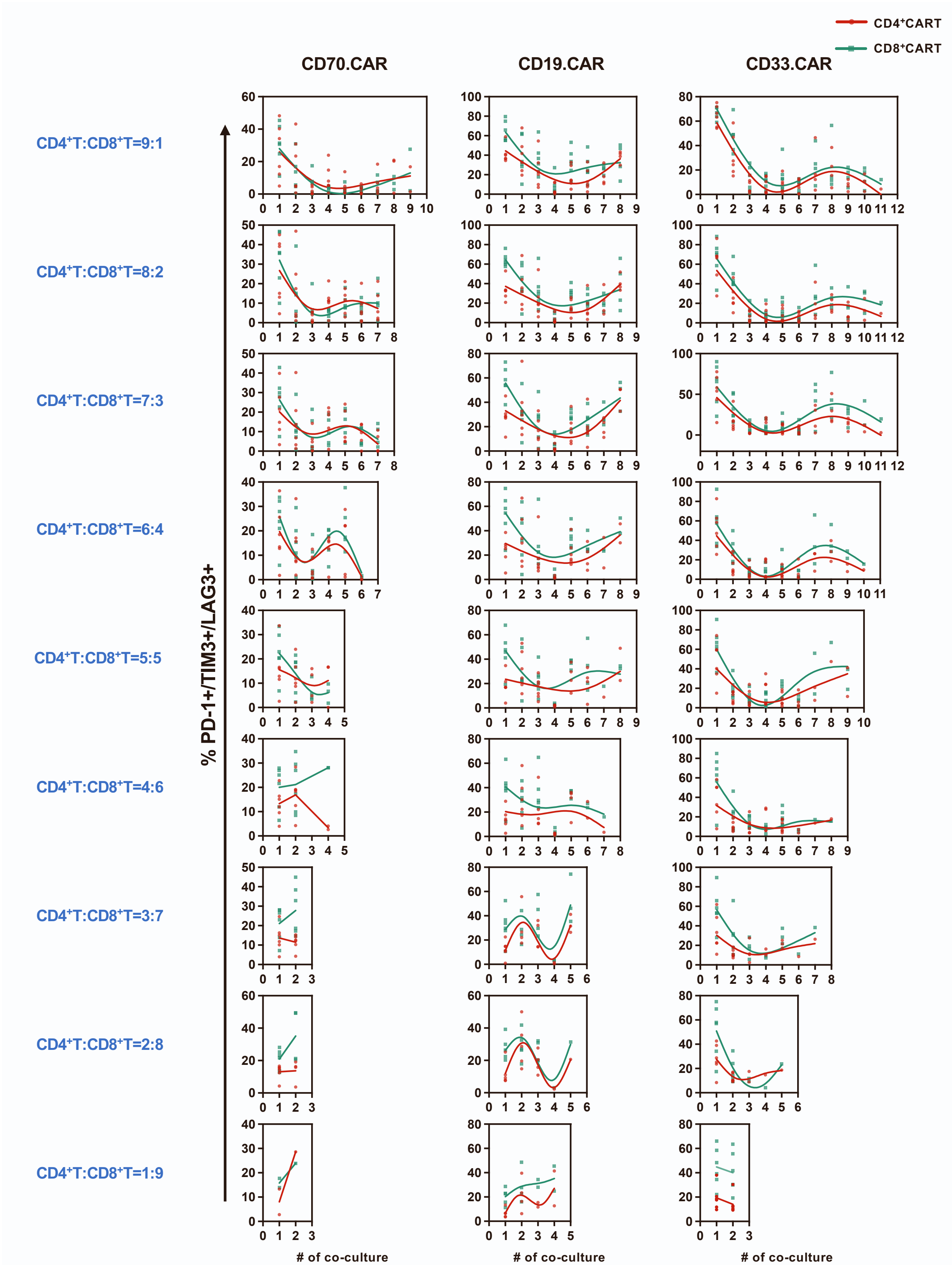

Figure S10. Expression of surface molecules associated with T-cell exhaustion on CD4^{+} and

CD8$^+$ CART cells during long-term coculture assays.

Expression of inhibitory receptors (PD-1+/TIM-3+/LAG-3+) on CD4$^+$ and CD8$^+$ CD70.CARTs, CD19.CARTs and CD33.CARTs from 6 different healthy donors at the end of each co-culture with their respective target cells as determined by flow cytometry. CARTs were added to the co-culture at the indicated CD4$^+$/CD8$^+$ ratios. Every data point displays the percentage of CD4$^+$ and CD8$^+$ CART cells expressing PD-1, TIM-3 and LAG-3 from every donor at each time point. The non-linear fit curve represents the fit value.

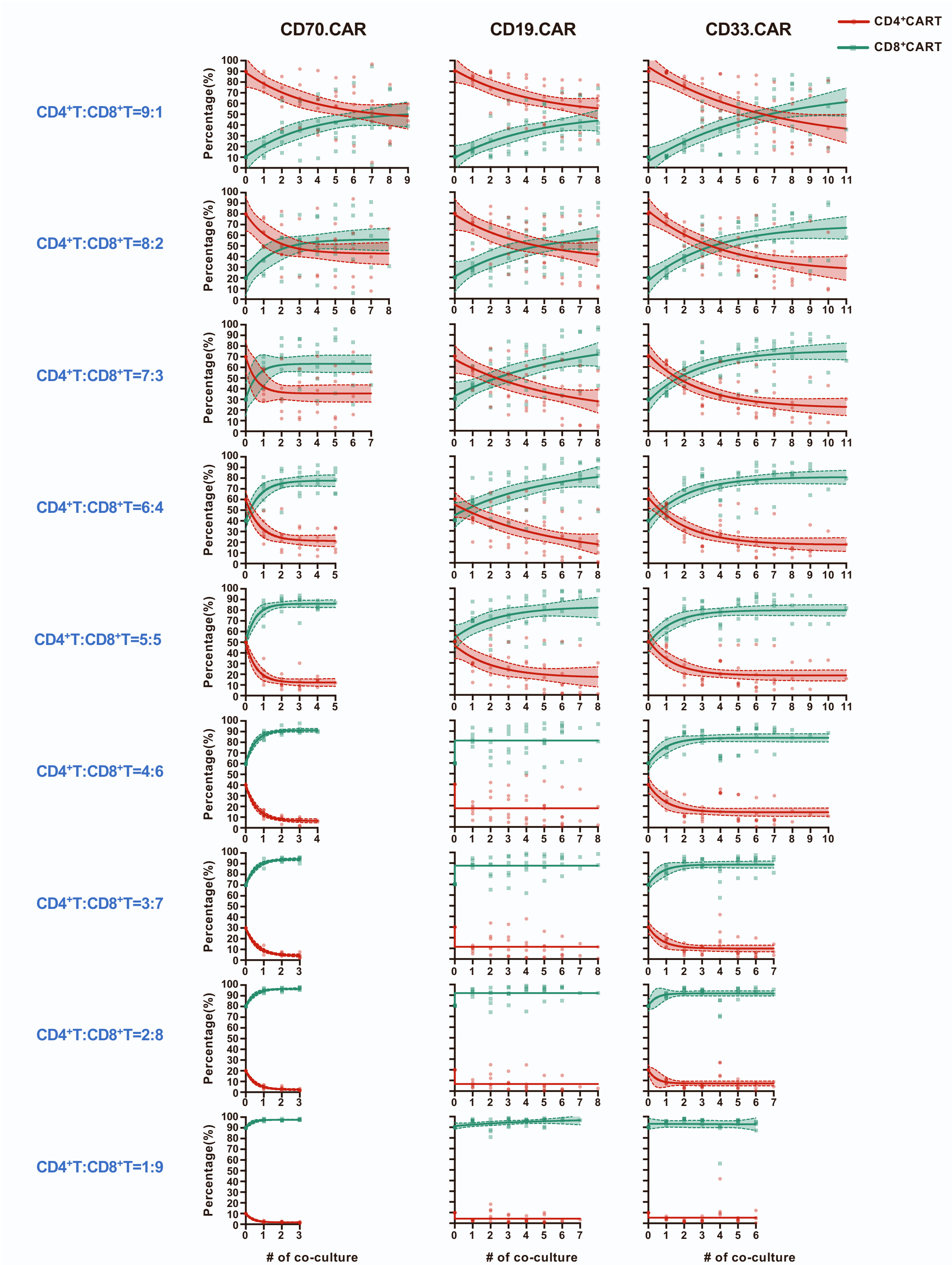

Figure S11. Changes of the CD4⁺/CD8⁺ T-cell composition of CART products during serial

coculture assays.

CD70.CART, CD19.CART and CD33.CART products from 6 healthy donors containing the indicated ratios of $CD4^+$ and $CD8^+$ CARTs were co-cultured with their appropriate target cells and the percentage of $CD4^+$ and $CD8^+$ CARTs for each donor was determined at the end of each co-culture by flow cytometry. Each individual datapoint displays the percentage of $CD4^+$ and $CD8^+$ CART cells of every donor at each time point. The non-linear fit curve represents the fit value and the dotted line represents the 95% confidence interval (CI).

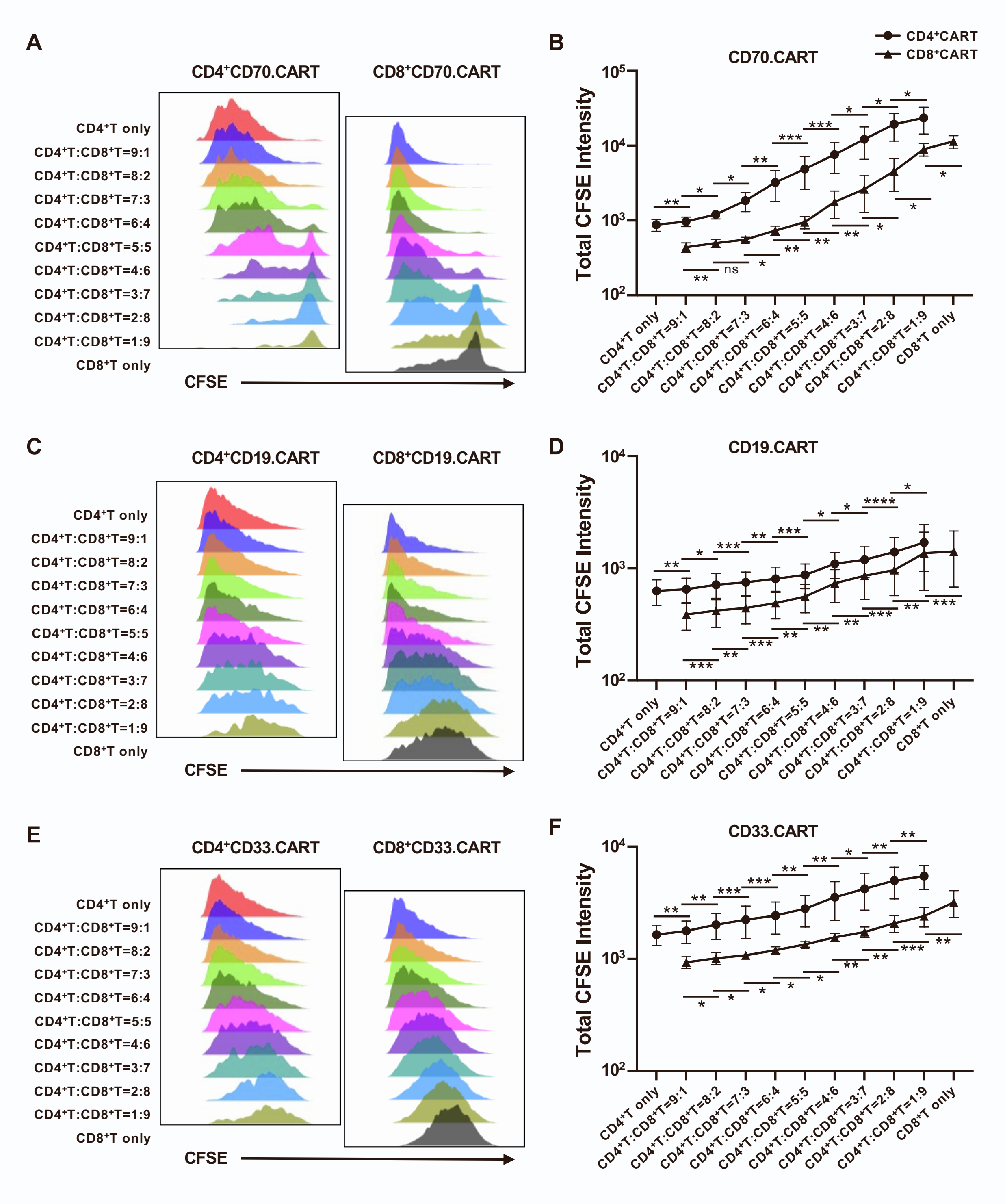

Figure S12. Proliferation rates of CD4$^+$ and CD8$^+$ CART cells depending on the CD4$^+$/CD8$^+$ composition upon antigen stimulation.

(A-B) Representative histograms (A) and statistical analysis (B) of the proliferation rate of CD4$^+$ and CD8$^+$ CD70.CART products containing the indicated CD4$^+$/CD8$^+$ ratios after a 5-day stimulation with irradiated Molm-13 cells as determined by CFSE staining. (C-D) Representative histograms (C) and statistical analysis (D) of the proliferation rate of CD4$^+$ and CD8$^+$ CD19.CART

products containing the indicated CD4$^+$/CD8$^+$ ratios after a 5-day stimulation with irradiated Nalm-6 cells as determined by CSFE staining. (E-F) Representative histograms (E) and statistical analysis (F) of the proliferation rate of CD4$^+$ and CD8$^+$ CD33.CART products containing the indicated CD4$^+$/CD8$^+$ ratios after a 5-day stimulation with irradiated HL-60 cells as determined by CSFE staining. (n = 4, paired t test, data represent mean ± SD, *P < 0.05; **P < 0.01; ***P < 0.001; ns, not significant).

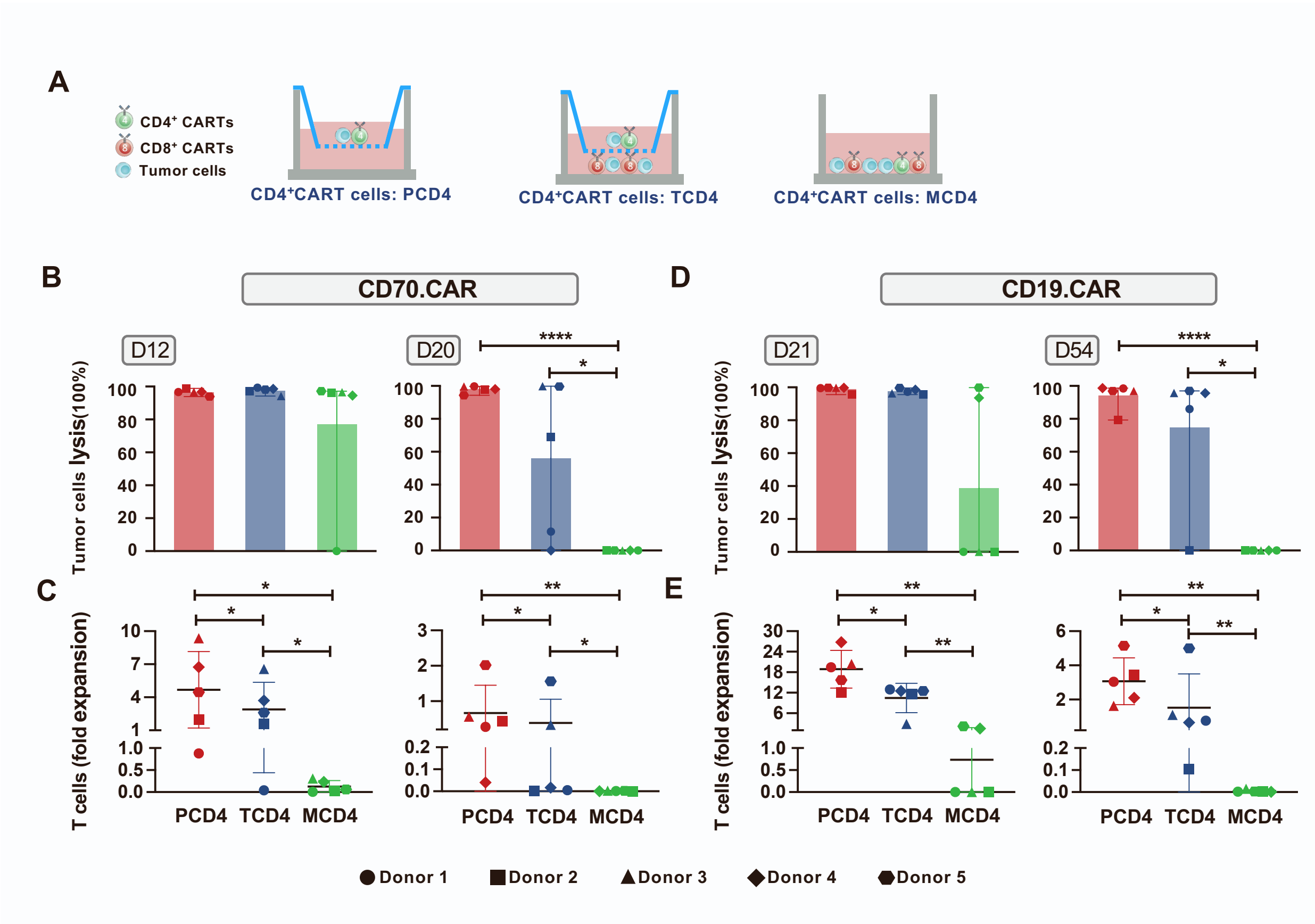

Figure S13. CD4$^+$ CART cell functionality is impaired by cell-to-cell interaction with CD8$^+$ CART cells.

(A) Experimental setup of the serial co-culture assay in the Transwell cell-culture device. CD70.CARTs and CD19.CARTs were co-cultured with Molm-13 or Nalm-6 cells at an E:T ratio of 1:1 and fresh tumor cells were added every 3 (CD19.CARTs) or every 4 days (CD70.CARTs) to both the top and bottom chambers. A fixed number of cells were added each time to maintain the initial predefined E:T ratio. Once the cells expanded beyond the spatial capacity in both chambers, they were transferred to larger inserts and wells to accommodate the increased volume. CD4$^+$ CARTs (in the upper chamber) separated from CD8$^+$ CARTs by the semipermeable membrane are indicated as TCD4, CD4$^+$ CARTs cultured in the upper chamber without any CD8$^+$ CARTs are indicated as PCD4 and CD4$^+$ CARTs that were co-cultured in the presence of CD8$^+$ CARTs are indicated as MCD4. The absolute tumor and T cell numbers at the end of each co-culture were determined by flow cytometry using CountBright counting beads. The T cell proliferation was calculated as the cumulative fold expansion based on the initial cell count. (B)

Tumor cell killing and (C) T-cell proliferation of CD70.CARTs on day 12 and day 20 of co-culture. The T cell proliferation was calculated as the cumulative fold expansion based on the initial cell count. (D) Tumor cell killing and (E) T-cell proliferation of CD19.CARTs on day 33 and day 54 of co-culture. (*n* = 5, paired t test, data represent mean ± SD, *P < 0.05; **P < 0.01; ****P < .0001).

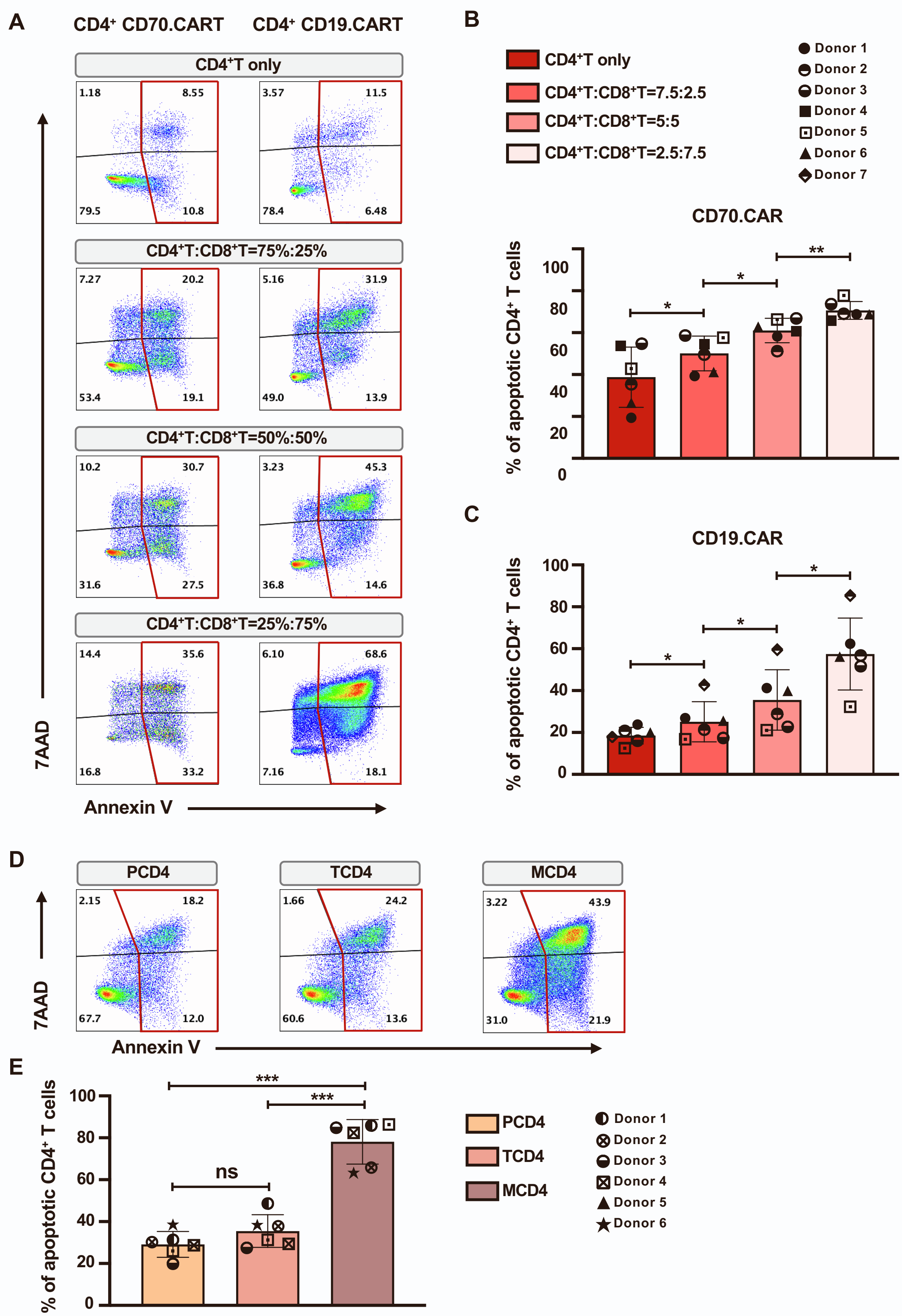

Figure S14. Induction of apoptosis in CD4⁺ CARTs upon interaction with CD8⁺ CART cells.

(A) Representative dot plots illustrating the percentage of apoptotic $CD4^+$ CD70.CARTs and CD19.CARTs of CART products containing the indicated $CD4^+/CD8^+$ ratios upon antigen stimulation in coculture assays as determined by Annexin V/7-AAD staining on day 8 for the CD70.CART model and day 12 for the CD19.CART model. CD70.CARTs and CD19.CARTs were cocultured with Molm-13 and Nalm-6 cells, respectively, at an E:T ratio of 1:3 and fresh tumor cells were added every 4 days (CD70.CARTs) and 3 days (CD19.CARTs) respectively. Apoptotic cells were identified as Annexin V+. (B) Quantitative analysis of apoptotic $CD4^+$ CD70.CARTs upon antigen stimulation. (C) Quantitative analysis of apoptotic $CD4^+$ CD19.CARTs upon antigen stimulation. (D) Representative dot plots illustrating the percentage of apoptotic $CD4^+$ CD19.CARTs harvested from the Transwell co-culture assay upon repetitive antigen stimulation with Nalm-6 cells every three days as determined by Annexin V/7-AAD staining on day 15. $CD4^+$ CARTs (in the lower chamber) separated from $CD8^+$ CARTs by the semipermeable membrane are indicated as TCD4, $CD4^+$ CARTs cultured in the lower chamber without any $CD8^+$ CARTs are indicated as PCD4 and $CD4^+$ CARTs that were co-cultured in the presence of $CD8^+$ CARTs are indicated as MCD4. (E) Bar graphs illustrating the percentage of apoptotic $CD4^+$ CARTs cell harvested from the Transwell co-culture assay. ($n = 6$, paired t test, data represent mean ± SD, $^*P < 0.05$; $^{**}P < 0.01$; $^{***}P < 0.001$).

A

	Anti-CD19 CD4 CART	Anti-CD19 CD8 CART	Anti-CD33 CD4 CART	Anti CD33 CD8 CART	Nalm-6	HL-60
Pure CD4 Anti-CD19	+				+	
Isospecific Anti-CD19	+	+			+	
Heterospecific Anti-CD19	+			+	+	
Dual Heterospecific Anti-CD19	+			+	+	+
Pure CD4 Anti-CD33			+			+
Isospecific Anti-CD33			+	+		+
Heterospecific Anti-CD33		+	+			+
Dual Heterospecific Anti-CD33		+	+		+	+

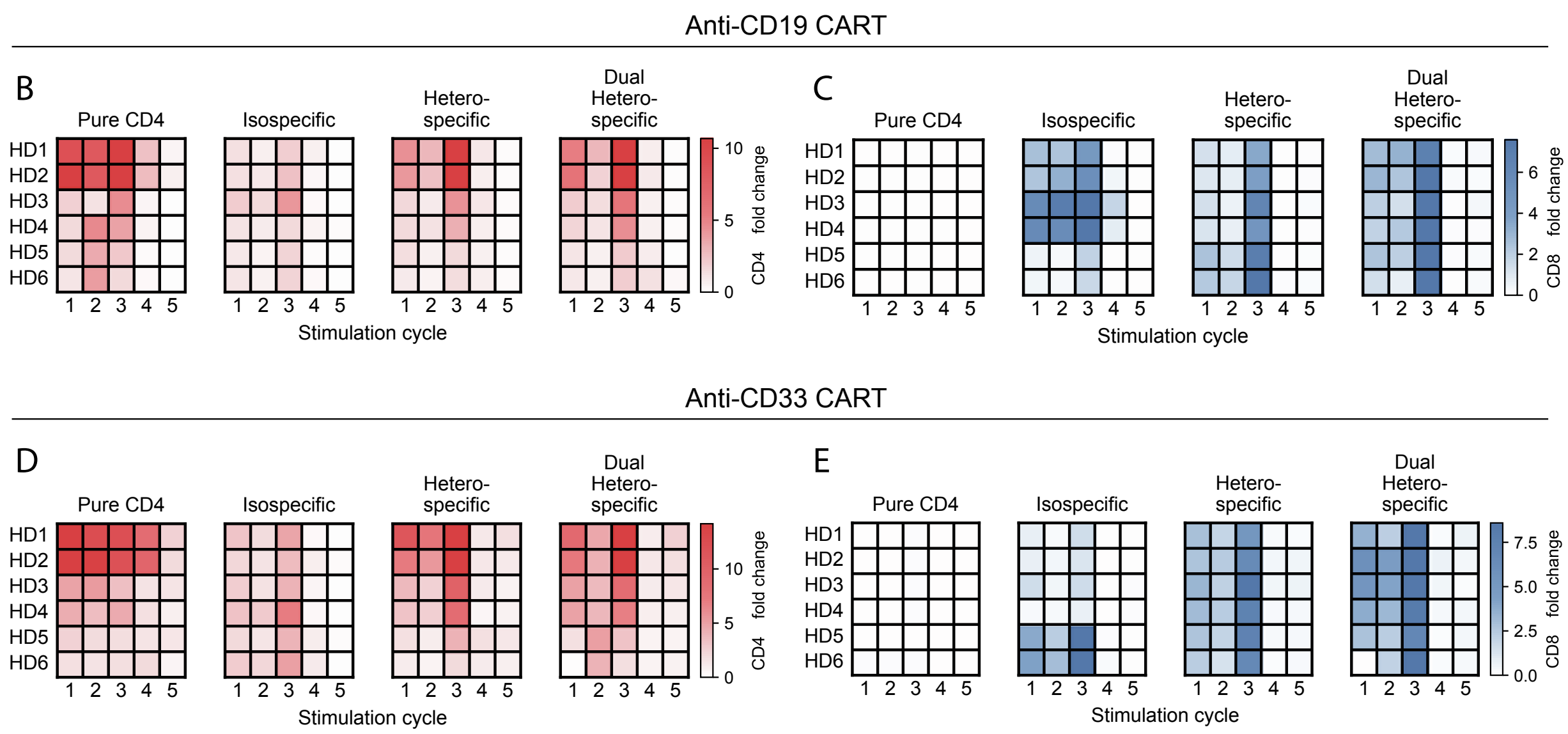

Figure S15. Dual heterospecific co-culture system identifies competitive antigen access as the principal mechanism of CD8$^+$ CART cell-mediated impairment of CD4$^+$ CART cell function. (A) Overview of the cellular and tumor cell composition of each co-culture condition. "+" denotes the presence of the respective component. The experiment comprises two parallel arms: an anti-CD19 arm using Nalm-6 as the primary tumor target and an anti-CD33 arm using HL-60. In heterospecific conditions, CD8$^+$ CART cells express an anti-CD33 CAR directed against an antigen absent from Nalm-6 and therefore receive no cognate stimulation. In dual heterospecific conditions, CD8$^+$ anti-CD33 CART cells are supplemented with HL-60 cells as a second tumor target, restoring productive CD8$^+$ engagement while maintaining segregation from the CD4$^+$-targeted antigen. (B, C) Serial co-culture assays of anti-CD19 CART cells under Pure CD4, Isospecific, Heterospecific, and Dual Heterospecific conditions. CART cells were co-cultured with Nalm-6 cells at an effector-to-target (E:T) ratio of 1:1, with fresh tumor cells added every 3 days.

The absolute tumor and T cell numbers at the end of each co-culture were determined by flow cytometry using CountBright counting beads. Heatmaps display individual donor $CD4^+$ (B) and $CD8^+$ (C) CART cell fold expansion relative to initial seeding across five successive stimulation cycles (columns) for six healthy donors (HD1–HD6, rows). Single-gradient heatmap of $CD4^+$ T cell proliferation (B): red, highest fold expansion; white, no expansion. Single-gradient heatmap of $CD8^+$ T cell proliferation (C): blue, highest fold expansion; white, no expansion. (D, E) Serial co-culture assays of anti-CD33 CART cells under the same four conditions, with HL-60 as the primary tumor target. Heatmap layout and colour scales as in (B) and (C), respectively.
